# Neuroregeneration of injured peripheral nerve by fraction B of catfish epidermal secretions through the reversal of the apoptotic pathway and DNA damage

**DOI:** 10.3389/fphar.2023.1085314

**Published:** 2023-01-16

**Authors:** Taiba A. Al-Arbeed, Waleed M. Renno, Jassim M. Al-Hassan

**Affiliations:** ^1^ Department of Anatomy, Faculty of Medicine, Kuwait University, Kuwait City, Kuwait; ^2^ Department of Biological Sciences, Faculty of Science, Kuwait University, Kuwait City, Kuwait

**Keywords:** neuroprotection, neuroregeneration, apoptosis, nerve injury, catfish fraction B

## Abstract

**Introduction:** Crush injuries occur from acute traumatic nerve compression resulting in different degrees of neural damage leading to permanent functional deficits. Recently, we have shown that administration of Fraction B (FB) derived from catfish epidermal secretions accelerates healing of damaged nerve in a sciatic nerve crush injury, as it ameliorates the neurobehavioral deficits and enhances axonal regeneration, as well as protects spinal neurons and increases astrocytic activity and decreasing GAP-43 expression. The present study aimed to investigate the role of FB treatment on the apoptotic pathway in the neuroregeneration of the sciatic nerve crush injury.

**Methods:** Male Wistar rats were randomly assigned into five groups: (I) SHAM, (II) CRUSH, (III) CRUSH + (1.5 mg/kg) FB, (IV) CRUSH + (3 mg/kg) FB, and (V) CRUSH + (4.5 mg/kg) FB. Rats underwent sciatic nerve crush surgery, followed by treatment with FB administered intraperitoneally (IP) daily for two weeks and then sacrificed at the end of the fourth week.

**Results:** FB improved the recovery of neurobehavioral functions with a concomitant increase in axonal regeneration and neuroprotective effects on spinal cord neurons following crush injury. Further, FB enhanced Schwann cells (SCs) proliferation with a significant increase in myelin basic protein expression. FB-treated animals demonstrated higher numbers of neurons in the spinal cord, possibly through ameliorating oxidative DNA damage and alleviating the mitochondrial-dependent apoptotic pathway by inhibiting the release of cytochrome c and the activation of caspase-3 in the spinal cord neurons.

**Conclusion:** FB alleviates the neurodegenerative changes in the lumbar spinal cord neurons and recovers the decrease in the neuronal count through its anti-apoptotic and DNA antioxidative properties.

## Introduction

Fine structural studies indicated that the epidermis of *Ariid* catfish (*Arius bilineatus Val*.) consists of two different types of secretory glandular cells; goblet (mucous) cells and club cells (Whitear and Mittal, 1983). The goblet cells produce slippery lubricating mucus consisting of high molecular weight glycoproteins. The club cells produce gel-like proteinaceous secretions composed predominantly of 85% proteins, 13.4% lipids, and small amounts (1.6%) of carbohydrate and nucleic acid components when catfish are threatened or injured (Al-Hassan et al., 1982). However, these gel-like proteinaceous secretions also give the catfish protection and resistance to diseases (Pinky and Mittal, 2008).

Marked differences in chemical and physical properties are exhibited between the threat-induced epidermal gel secretions from the catfish *Arius bilineatus, Val*. And the other three *Ariid* catfish species found in the Arabian Gulf and their normal epidermal mucous secretions. The threat-induced epidermal gel secretions are prominent in thickness, whitish in color, and very viscous. Their mucus is transparent and watery. The epidermal secretions are released first. The mucus is only seen after the removal of the epidermal secretions. The carbohydrates within the gel-like secretions are not typical of the carbohydrates in mucus. Mucus is generally made up of polysaccharides with minor protein components. The predominant contents of carbohydrates within the catfish epidermal secretions are galactosamine, glucosamine, and galactose (Al-Hassan et al., 1985). Proteins in the epidermal secretions of the catfish aggregate to form insoluble gel-like material in the seawater (Al-Hassan et al., 1987). The more significant portion of the aggregate comprises alpha-helical protein structures that provide the skeleton for aggregation of the other proteins and lipids. The alpha-helical proteins are insoluble in most common solvents but soluble in 80% formic acid (Al-Hassan et al., 1982; Al-Hassan et al., 1986).

Recent results at MD Anderson Cancer Center in Houston, TX, have demonstrated that different lipid-soluble fractions separated from catfish skin preparations (CSP) have anti-inflammatory and anti-proliferative effects against prostate, pancreatic, lung, skin, and liver cancer cell lines (Yang et al., 2016; Al-Hassan et al., 2019; Al-Hassan et al., 2021). Our collaborators at Sick Children Hospital Research Institute, Toronto, have established that lipid fractions and pure lipid components from skin secretions have high activity against leukemia K562 synergistically with Gleevec (Al-Hassan et al., 2020). In addition, recently, we proved that Fraction B has a potent effect against human (Panc-1) and mouse (Panc-02) pancreatic cancer cells (Al-Hassan et al., 2019) and breast MDA MB-231 and leukemic K-562 cancer cell lines in a dose-dependent fashion (Al-Hassan et al., 2021). FB treatment also accelerated wound healing created by a transdermal punch biopsy of the rat skin (Al-Hassan et al., 2020). Further, topical application of preparations from CSP exhibited acceleration of wound healing in animals and humans (Al-Hassan et al., 1983; Al-Hassan et al., 1991; Al-Hassan et al., 2020), potent anti-inflammatory and healing properties as demonstrated by previous preliminary clinical trials for healing of non-healing diabetic foot ulcers, chronic back pain and other neurological disorders (Al-Hassan, 1990; Renno et al., 2021). These preliminary trials indicated that CSP had a noticeable effect on neuropathy in the diabetic foot and potent healing effects on chronic back and joint pain.

Sciatic nerve crush injury is the most commonly studied nerve injury and has been used as a model to test the mechanisms of controlling peripheral nerve regeneration (Myckatyn and MacKinnon, 2004; Schiaveto de Souza et al., 2004). Although sciatic nerve injuries in humans are rare due to the deep anatomical location within the lower extremity, the animal model provides information regarding recoveries of particular nerve types and their potential for recovery, as new axons attempt to reach endoneurial tubules (Menorca et al., 2013). Also, the nerve crush model represents mild forms of nerve compression injuries or neuropathy, such as that resulting from diabetes and myelin-related neurodegenerative diseases. Before nerve fibers regeneration can occur, a sequence of degenerative processes must occur depending on the type of nerve fiber affected, either motor or sensory neurons and myelinated or unmyelinated fibers, the type and severity of the injury, and consequences of deficits in axonal transport or demyelination. Also, several factors influence the survival of neuronal cells following severe peripheral nerve injury. It is believed that the neuronal cells are unreplaceable after death, resulting in poor regeneration and loss of sensory and motor functions (Wiberg et al., 2003; Schenker et al., 2006). The balance between regenerative and cell death signals activated after injury determines the survival or death of the neuronal cell (Nunez and del Peso, 1998). Different intrinsic and extrinsic cellular signaling pathways regulate this balance, including pro-apoptotic and pro-survival signals. Neuronal cell death is regulated by apoptosis, an active process distinguished morphologically by cell shrinkage, chromatin condensation, internucleosomal DNA cleavage, reorganization of the cytoskeleton, and apoptotic bodies (Adams and Cory, 1998). After peripheral nerve injury, the changes in electrical activity, neurotoxic inflammatory products, and loss of neurotrophic support, lead to apoptosis of neuronal cells (Fu and Gordon, 1997). In this study, we propose the following hypothesis: FB derived from CSP has anti-apoptotic and neuro-regenerative effects on the injured peripheral nerve, which may enhance neuronal survival and support axonal regeneration by modulating apoptotic and oxidative cell damage.

## Materials and methods

### Animals

Adult male Wistar rats (11–12 weeks old) weighing 200–250 g were used in this study. The animals provided by the Animal Resources Center of the Faculty of Medicine at Kuwait University were housed in pairs in plastic cages with sawdust bedding and maintained on a 12/12-h light/dark cycle and 21°C–25°C temperature. The animals were given free access to food and water *ad libitum*. Animal experiments followed the Animal Ethics Committee of Kuwait University Health Sciences Center procedures, and the study was carried out under the US guidelines (NIH Publication #85-23, revised in 1985). All efforts were made to minimize the number of animals used in the study and their suffering.

### Sciatic nerve crush surgical procedure

The animals (n = 40) were randomly divided into five groups with equal numbers (n = 8/group): I: sham-operated rats; II: crushed sciatic nerve rats; III: crushed sciatic nerve rats treated with 1.5 mg FB/kg animal body weight; IV: crushed sciatic nerve rats treated with 3 mg FB/kg body weight; V: crushed sciatic nerve rats treated with 4.5 mg/kg body weight. The FB-treated animals were administered with intraperitoneal (IP) injections of FB according to the dose designed for each group once a day for 14 days starting 1 h after sciatic nerve crush injury. The sciatic nerve crush surgical procedure was performed as previously described (Renno et al., 2013; Renno et al., 2017; Renno et al., 2021). Briefly, the animals were deeply anesthetized for surgery using a mixture of Ketallar® 62.5 mg/kg [Tekam® 50 (Ketamine HCl), Hikma Pharmaceuticals, Amman/Jordan] and Rompun® 3.2 mg/kg [2% Xylazine HCl; Bayer GMP] *via* intraperitoneal injection. The right hindquarter was shaved and then sterilized with 70% alcohol. After preparation, a 2 cm incision was made in the skin of the lower right thigh. The skin was then dissected from the underlying musculature, and the gluteus maximus muscle and the anterior head of the biceps femoris muscle were teased apart with fine scissors and the sciatic nerve was exposed. The nerve was freed gently from the surrounding connective tissue and crushed at 10 mm from the sciatic notch for 60 s using a micro mosquito (Strait, 12.5 cm, Serrated 0.4 × 0.8 mm tip, World Precision Instruments, Inc. FL, United States). The nerve was then examined to ensure the intact epineural sheath but axotomized as indicated by its translucent appearance. The skin wound was closed with clips after repositioning the muscles. Loss of motor and sensory function in the operated right limb was noticed after the completion of the crush. Also, loss of foot withdrawal and vocalization when pinching the digits of the operated right limb was noted as a loss of motor and sensory function. The sciatic nerve of the sham group was exposed, but no crush injury was done, and then the muscles were repositioned, and the skin was closed using autoclips. After 7 days, the autoclips were removed from all animals using autoclip remover.

As reported previously, none of the animals died during the experiment (Renno et al., 2021). The animals appeared healthy and grew normally in size and weight, comparable to the normal untreated matching group.

### Collection of samples at sea and fractionation of CSP from the skin of the catfish

Catfish were caught on baited hooks online in the Kuwaiti territorial waters as previously described (Al-Hassan et al., 1982). The caught fish was washed thoroughly with seawater to remove contaminants on the skin, leaving the skin-covering proteinaceous epidermal gel clean. The catfish gel prepared from the skin (skin secretions, CSP) was collected by gently scraping the fish with a blade. The collected material was frozen in dry ice and stored at −80°C until use. The gel-like material was fractionated to yield FB containing proteins and lipids utilizing the procedures described in the previously published two patent applications (US patents # 10,568,915 B1 and # 8,551,532 B2).

### Protein assay in FB

The protein content in FB was assayed with the Coomassie Blue method (Bradford, 1976). The protein concentration was calculated from the standard curve at λ 595 nm generated with bovine albumin), and the linear curve was used to calculate the total proteins in FB. The obtained concentration of the stock FB was used to provide the desired concentration to be IP injected into the experimental animals (1.5 mg; 3.oo mg; 4.5 mg protein/kg animal body weight).

### Assessment of motor and sensory functional recovery

Two well-trained individuals handled the rats and evaluated them for motor and sensory activities. Behavioral tests were carried out for 2 weeks before the initiation of the study to obtain baseline data. After sciatic nerve surgery, all the behavioral tests were performed once a week and distributed on different days from the first-week post-surgery until the day of sacrifice. All behavioral tests were conducted two times with 20 min intervals for each rat, and the average of the two readings was used for statistical data analysis. Investigators were blinded to all treatments in all experiments Renno et al., 2017; Renno et al., 2021).

Assessment of motor tests recovery was conducted as described in our previous studies using foot position, toe spread, extensor postural thrust (EPT), hopping, and rotarod tests (Renno et al., 2017; Renno et al., 2021). EPT was measured by calculating the percentage of the functional motor deficit; thus, a high value indicates a poor outcome (Luis et al., 2007; Renno et al., 2017; Renno et al., 2021). The hopping test was done to test several integrated sensory and motor functions by moving the rat’s leg laterally above a horizontal surface. The test score indicated whether the rat hopped on the table surface (1 for hopping, 0 for no hopping) (Thalhammer et al., 1995). The rotarod test was done using the rotarod device (47750—Rat Rotarod NG, Ugo Basile SRL, Varese—ITALY) to evaluate the motor coordination of the rat. The starting speed of rotarod was adjusted to 5 rpm with an acceleration rate of 20 rpm/min, and the maximum speed was 45 rpm. The rat was held by the tail facing away from the direction of rotation and placed on the rotating rod, so it had to walk forward to stay upright. Acceleration was started after 10 s of placing the rat, allowing the rat to be trained. The speed was noted once the rat fell off. Falling before 5 s because of poor experimenter placing was not recorded. The rat scored 4 rpm when it failed to grip in 10 s (Deacon, 2013).

Assessment of sensory functions was conducted as previously described using hotplate analgesia and tail-flick neurobehavioral tests (Renno et al., 2017; Renno et al., 2021). The tail-flick test was used to test the spinally mediated nociceptive thresholds that were carried out using an Analgesia Meter Apparatus (Model 7,360, UGO Basile SRL, Italy). The animal was gently restricted by hand, and radiating heat (IR Intensity 20) was passed to the proximal 2/3 part of its tail. The amount of time animal spent moving its tail away from the heat was recorded with 30 s cut-off time to avoid tissue damage. Thermal nociception was measured by a modified hot plate test (51°C; 35100—Hot Cold Plate, Ugo Basile SRL, Italy). The withdrawal response was defined as the withdrawal of the hind paw and was characterized as a paw flick and recorded to the nearest 0.1 s; a standard 35 s cut-off latency was used to avoid tissue damage. All sensory tests were repeated two times at a gap of 30 min.

### Immunohistochemical, histological, and biochemical analysis

#### Tissue preparation for immunohistochemistry

All animal groups were sacrificed on the 28th-day post sciatic nerve crush injury using CO_2_ asphyxiation. Sciatic nerves and lumbar spinal cords were dissected, removed, and immersed in a mixture of 4% paraformaldehyde and 0.1% glutaraldehyde in 0.1 M phosphate buffer, pH 7.4 overnight at 4°C. The next day, tissues were placed in a plastic cassette and manually processed through different steps, starting with the dehydration step as previously described. The tissues were then cleared in xylene, followed by infiltration in wax. In the end, the tissues were embedded in paraffin and kept to solidify at room temperature. Seven μm thick paraffin sections of the spinal cord were cut using rotary microtome, mounted on Poly-l-lysine coated slides, and kept overnight for drying. Every five sections per group of the spinal cord were stained for Cresyl Violet staining and immunohistochemistry Renno et al., 2017; Renno et al., 2021).

#### Immunohistochemistry for NeuN, c-Jun, c-Fos, and 8-OHDG in spinal cord tissues

The spinal cord sections were deparaffinized by heating at 60°C, washed in xylene (I) and xylene (II), rehydrated through a graded series of ethanol and washed in distilled water. Endogenous peroxidase activity was quenched by incubating the sections with 3% hydrogen peroxide (H_2_O_2_) for 15 min, followed by incubation with 50 mM glycine +0.1% sodium borohydride for 30 min, and then washed with PBS for 5 min. After that, the sections were treated with 5% normal goat serum (Sigma, G9023) for 1 h to block the non-specific binding of antibodies. The sections were then incubated with primary antibodies [1:200] Anti-NeuN (Millipore, MAB377), Anti-8-OHdg (Santa Cruz Biotechnology, sc-66036), Anti-c-Jun (Santa Cruz Biotechnology, sc-1694), and Anti-c-Fos (Santa Cruz Biotechnology, sc-253) for overnight at 4°C. The sections were washed for 5 min with PBS three times and treated with biotinylated goat anti-mouse IgG (Vector Laboratories, BA-9200) and biotinylated goat anti-rabbit IgG (Vector Laboratories, BA-1000) antibody diluted in 1% normal goat serum for 30 min at room temperature. Slides were then washed in PBS three times for 5 min and treated with Avidin-Biotin Complex (Vector Laboratories, PK-6200) in conjugation with 0.1% Tween20 for 30 min at room temperature. Next, sections were exposed to 3-diaminobenzidine (DAB) (Vector Laboratories, SK-4105) under the microscope for 30 s or until the required brown color developed. The slides were then washed with distilled water and counterstained with hematoxylin for 1 min, followed by 5 min bluing under tap water. The slides were then dehydrated by passing through graded series of alcohol and cleared with xylene (I) and xylene (II). Finally, the slides were mounted with a coverslip using DPX mountant (Sigma, 44581) (Renno et al., 2017; Renno et al., 2021).

The number of immunolabeled neurons in the spinal cord’s ventral and dorsal grey horns was counted using Cell Sens Dimension software. From each rat, ten sections were selected for neuron quantification. The spinal cord region under analysis was focused at ×40 magnification, and an image was transferred to a computer monitor with a high-resolution digital Nikon camera attached to an Olympus microscope (DP-72). The total number of neurons in the entire ventral and dorsal grey horns was counted. Slides were coded to avoid observers’ bias. The mean number of neurons per section was calculated for statistical analysis (Renno et al., 2017; Renno et al., 2021).

#### Cresyl violet staining

The spinal cord sections were deparaffinized (by heating at 60°C followed by dewaxing in xylene (I) and xylene (II)), rehydrated through a graded series of ethanol (absolute (I), absolute (II), 90%, 70%, 50%) and washed with distilled water. The tissue sections were then stained with 0.1% Cresyl violet for 15–20 min, followed by differentiation and dehydration through ascending grades of ethanol (70%, 90%, absolute (I), absolute (II)). In the end, the sections were cleared in xylene (I) and xylene (II) for 5 min, then covered with cover-slipped with DPX mountant (Sigma, 44581).

#### Whole-mount staining

As previously described, sciatic nerves were processed for the whole mount staining procedure (Dun and Parkinson, 2015). Briefly, after fixation, nerves were washed three times in PTX (1% Triton X-100 (Sigma, T9284) in phosphate-buffered saline (PBS)) for 10 min each time after the fixation. For nerve crush samples, the epineurium was separated after washing in PTX to guarantee antibody penetration. Nerves were incubated with blocking solution (10% fetal bovine serum (FBS) in PTX) at 4°C overnight. The next day, nerves were moved into primary antibodies in PTX containing 10% FBS. They were incubated for 48–72 h at 4°C with gentle rocking. Primary antibodies that were used for the study were S100β (1:100, Agilent Technologies, Z031101-2), neurofilament heavy chain (1:1000, Abcam, ab4680), myelin basic protein (1:100, Santa Cruz Biotechnology, SC-271524), and Ki67 (1:100, Abcam, ab15580). Once the incubation was done, nerves were washed three times with PTX for 15 min for each wash, followed by 6 h of washing in PTX with a change of PTX every 1 h at room temperature. Secondary antibodies (1:500, Abcam) and Hoechst dye (1:1000, Thermo Fisher Scientific, H3570) were diluted in PTX containing 10% FBS, followed by incubation with the nerves for 48 h at 4°C with rocking. Nerves were then washed three times each in PTX for 15 min, followed by 6 h of washing with PTX with changing the PTX each hour at room temperature, and were then rinsed overnight at 4°C without changing PTX. Clearance of nerves was done sequentially with 25%, 50%, and 75% glycerol (Sigma, G6279) in PBS between 12–24 h for each glycerol concentration. Mounting nerves in CitiFluor (Agar Scientific, AGR1320) were performed for confocal imaging following the clearance. Images were taken using Zeiss LSM 700 confocal microscope (Dun and Parkinson, 2015).

#### Application of terminal deoxynucleotidyl transferase Biotin-DUPT nick end labeling (TUNEL)

TUNEL procedure was done using an *insitu* death detection kit (fluorescein; Roche, 11684795910). The spinal cord sections were processed through dewaxing (by heating at 60°C followed by washing in xylene (1) and xylene (2)), rehydration through a graded series of ethanol (absolute (1), absolute (2), 95%, 70%) and washing in distilled water. Antigen unmasking reagent proteinase K (10–20 μg/ml in 10 mM Tris/HCl, pH 7.4–8) was directly applied to tissue sections for 15–30 min at 21–37°C, followed by twice rinsing with PBS. The TUNEL reaction mixture was prepared immediately before use by adding 50 μL enzyme solution to 450 μL label solution and mixing them well. Negative control slides were incubated with label solution instead of the TUNEL reaction mixture, while the positive control was incubated with a micrococcal nuclease or DNase I recombinant (3000 U/ml-3U/ml in 50 mM Tris-HCl, pH 7.5 mg/ml BSA) for 10 min at 15–25°C before labeling procedure. Next, the TUNEL reaction mixture was added to the slides. The slides were then incubated in a humidified atmosphere for 1 h at 37°C in the dark. This step was followed by three times rinsing the slides in PBS. Sections were then counterstained with the Hoechst solution. Samples were directly analyzed under a fluorescence microscope. Apoptotic cells were defined as those cells with TUNEL-positive nuclei that were excited at 488 nm wavelength, and their emission was recorded between 515–565 nm.

#### Western blotting

Western blot analysis was performed as described previously (Renno et al., 2017; Renno et al., 2021). Spinal cords and sciatic nerves were removed from animals (n = 5/group), immersed in liquid nitrogen, and kept at −80°C until use. Tissues were homogenized in a mixture of RIPA buffer (sc-24948), PMSF, Na orthovanadate, and protease inhibitor, with the temperature maintained at 4°C. Following homogenization, samples were centrifuged at 10,000xg for 30 min. The supernatant fluid was collected and the total protein concentration was then estimated. The samples were adjusted so as each spinal cord sample contained 50 μg protein and each sciatic nerve sample contained 75 μg protein. Sample, containing tissue lysate, deionized water, and an equal amount of 4x LDS Sample Buffer (Invitrogen, NP0007) and 10x Sample Reducing Agent (Invitrogen, NP0009) and 10 μL was used. The mixture was vortexed, boiled at 95ºC−100°C for 5 min, and then centrifuged at room temperature at 17,000 x g for 30 min. Mini-PROTEAN TGX™ precast gel (Bio-Rad, 4561096) was immersed in running buffer [50 ml 20X NuPAGE MES SDS running buffer (Novex, NP0002) + 950 ml deionized water]. Seven μl of reference marker (Novex, LC5800) was loaded into the first well of each gel. Equal amounts of protein (10 μL) of each sample were loaded in the remaining wells, and the running buffer was added to the electrophoresis’s outer chamber. Electrophoresis was then started with 200 V for about 45 min. The PVDF membrane (Thermo Fisher Scientific, 88520) was activated in methanol, distilled water, and transfer buffer, each for 10 min. The transfer sandwich was prepared, and the proteins were transferred from the gel to PVDF membrane for 1.5 h at 75 V in cold fresh 1X transfer buffer [50 ml of 20x NuPAGE transfer buffer (Novex, NP0006-1) + 100 ml methanol +850 ml deionized water]. Membranes were washed in PBS 3 times for 5 min and then blocked with 5% non-fat dry milk dissolved in Tween/Tris-buffered salt solution (TBST) for 30–60 min at room temperature. The membranes were then incubated with primary antibodies against the following proteins: Bax (sc-7480, Santa Cruz Biotechnology), Bcl-2 (sc-7382, Santa Cruz Biotechnology), caspase 3 (sc-56053, Santa Cruz Biotechnology), and cytochrome C (sc-13561, Santa Cruz Biotechnology) antibodies (1:500 dilution) at 4°C overnight. After incubation, the membranes were washed vigorously with a large amount of TBST and incubated with the anti-mouse IgG secondary antibody conjugated to horseradish peroxidase (sc-516102, Santa Cruz Biotechnology) for 1–2 h at room temperature on a shaker. After washing the membranes with TBST 3 times each for 5 min, they were stained with an ECL kit (GE Healthcare, RPN2109) for 1 min and then exposed to X-Ray photographic film (Santa Cruz Biotechnology, sc-201696) in a cassette. Following this, the X-Ray film was developed, then dried from water under a hot air fan. Actin (Sigma, A2228) was used as a loading control. All bands labeled with the Bax, Bcl-2, cytochrome C, and caspase 3 antibodies were scanned. Their densities were quantified by Densitometer GS-800 (Bio-Rad Quantity One 4.6.3) normalized with the actin band density.

#### Statistical analysis

All data were analyzed by one-way analysis of variance ANOVA followed by the Least Square Difference (LSD) comparison *post hoc* test. All statistical analysis were performed using Statistical Package for the Social Sciences (SPSS; version 21.0, IBM Corp.). *p* values less than 0.05 were considered statistically significant.

## Results

The clinical appearance of the right hind paws and walking activities were normal in the SHAM-operated group during the first-week post-surgery. This indicated that skin and muscle wounds did not impede the rat’s overall posture and gait. However, the right hind paws in the CRUSH and CRUSH-treated groups (1.5 mg/kg FB, 3 mg/kg FB, and 4.5 mg/kg FB) were flexed. These animals also showed difficulty standing on their hind paws throughout the first-week post-surgery. During the second week, partial weight-bearing was observed in the CRUSH and CRUSH + 1.5 mg/kg FB-treated rats. Observable improvements in the right hind paws’ appearance, weight-bearing, and wound closure were also observed in the 3 mg/kg FB and 4.5 mg/kg FB CRUSH-treated groups within until the day of sacrifice. No deformity or gross pathological alterations in all the organs were detected at the end of the experiment. These findings agree with our recent *in vivo* and *in vitro* studies that showed Fraction B has no toxic effect on the blood cells of the treated animals (Al-Hassan et al., 2021). In another study, normal healthy animals were treated daily with FB (IP) for 4 months, grew well, and on dissection, showed no physical abnormalities (data not shown).

### FB derived from catfish skin preparations enhances motor and sensory neurobehavioral functions recovery after sciatic nerve crush injury

**FIGURE 1:**
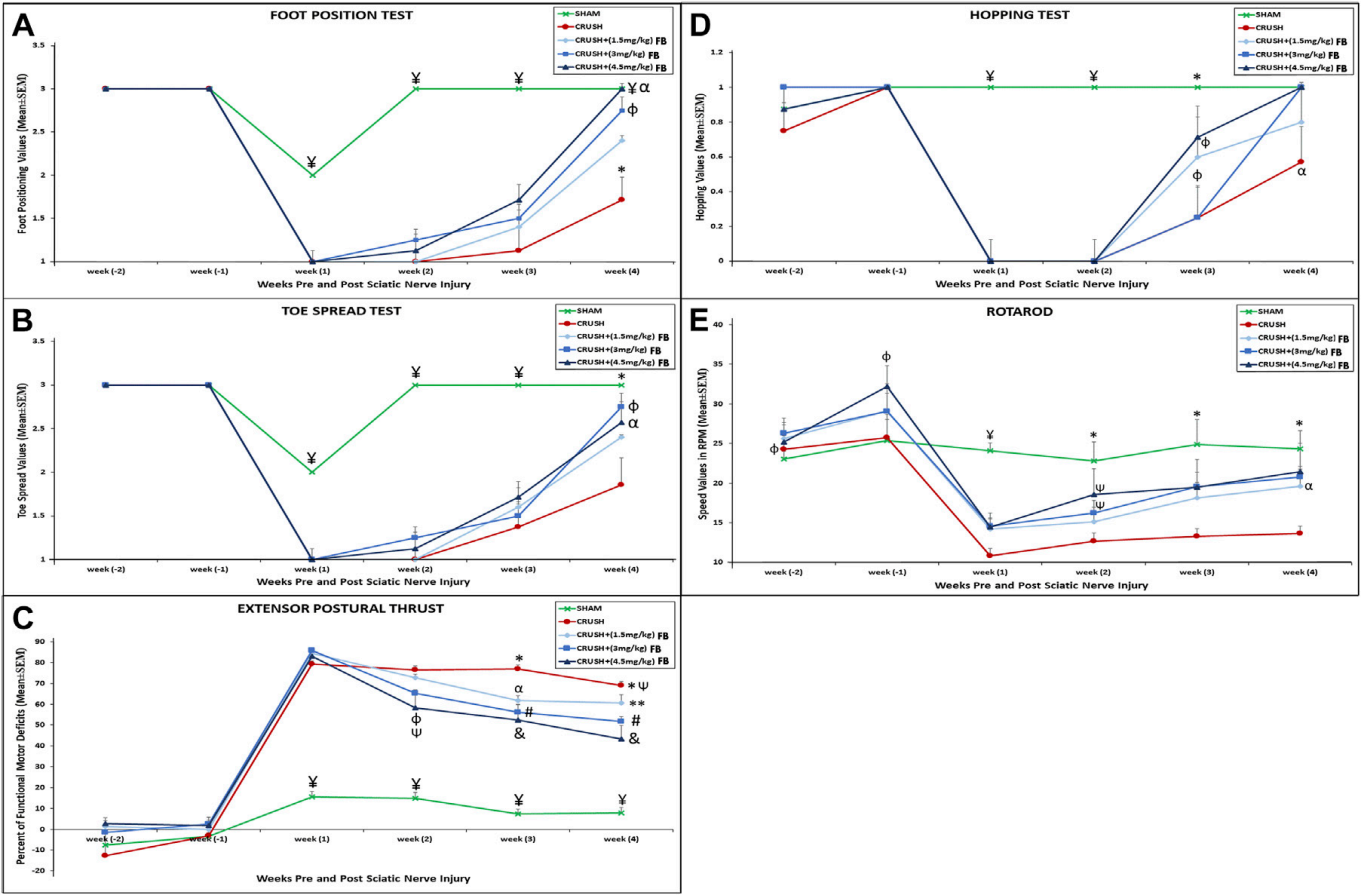
**(A)** Differences in the mean values for the foot position analysis for the experimental groups. ¥ indicates *p* < 0.001, SHAM vs. CRUSH, CRUSH+(1.5 mg/kg)FB, CRUSH+(3 mg/kg)FB and CRUSH+(4.5 mg/kg) FB; * indicates *p* < 0.001, CRUSH vs. SHAM, CRUSH+(3 mg/kg)FB and CRUSH+(4.5 mg/kg)FB; φ indicates *p* < 0.01, CRUSH+(1.5 mg/kg)FB vs. CRUSH+(3 mg/kg)FB and CRUSH+(4.5 mg/kg)FB; α indicates no significant difference between SHAM vs. CRUSH+(3 mg/kg)FB and CRUSH+(4.5 mg/kg)FB. **(B)** Differences in the mean values for the analysis of the spread outcome. The treated rats showed statistically significant clinical recovery following sciatic nerve injury in toe spread.¥ indicates *p* < 0.001, SHAM vs. CRUSH, CRUSH+(1.5 mg/kg)FB, CRUSH+(3 mg/kg)FB and CRUSH+(4.5 mg/kg)FB; * indicates *p* < 0.01, SHAM vs. CRUSH, and CRUSH+(1.5 mg/kg)FB; φ indicates *p* < 0.01, CRUSH+(3 mg/kg)FB vs. CRUSH and CRUSH+(3 mg/kg)FB; αindicates *p* < 0.05, CRUSH+(4.5 mg/kg)FB vs. CRUSH and CRUSH+(1.5 mg/kg)FB. **(C)** Evaluation of functional recovery as measured by EPT following crush injury and FB treatment. EPT was measured by calculating the percentage of the functional deficit; thus, a high value indicates a poor outcome. ¥ indicates *p* < 0.001, SHAM vs. CRUSH, CRUSH+(1.5 mg/kg)FB, CRUSH+(3 mg/kg)FB and CRUSH+(4.5 mg/kg)FB; * indicates *p* < 0.01, CRUSH vs. CRUSH+(3 mg/kg)FB; φ indicates *p* < 0.01, CRUSH+(4.5 mg/kg)FB vs. CRUSH; Ѱ indicates *p* < 0.05, CRUSH and CRUSH+(4.5 mg/kg)FB vs. CRUSH+(1.5 mg/kg)FB; αindicates *p* < 0.05, CRUSH+(1.5 mg/kg)FB vs. CRUSH; # indicates *p* < 0.01, CRUSH+(3 mg/kg)FB vs. CRUSH; ** indicates *p* < 0.01, CRUSH+(1.5 mg/kg)FB vs. CRUSH+(4.5 mg/kg)FB; & indicates *p* < 0.001, CRUSH+(4.5 mg/kg). **(D)** Differences in the mean values of the hopping test outcome among the experimental animal groups. ¥ indicates *p* < 0.001, SHAM vs. CRUSH, CRUSH+(1.5 mg/kg)FB, CRUSH+(3 mg/kg)FB and CRUSH+(4.5 mg/kg)FB; * indicates *p* < 0.01, SHAM vs. CRUSH, and CRUSH+(3 mg/kg)FB; φ indicates *p* < 0.05, CRUSH+(1.5 mg/kg)FB and CRUSH+(3 mg/kg)FB vs. SHAM; αindicates *p* < 0.05, CRUSH vs. SHAM, CRUSH+(3 mg/kg)FB and CRUSH+(4.5 mg/kg)FB. **(E)** The rotarod test was performed to evaluate motor coordination and motor learning. The treated groups showed marked improvement in this test compared to their performance on the first day of testing. In contrast, the CRUSH group did not show an improvement in the rotarod test. ¥ indicates *p* < 0.001, SHAM vs. CRUSH, CRUSH+(1.5 mg/kg)FB, CRUSH+(3 mg/kg)FB and CRUSH+(4.5 mg/kg)FB; φ indicates *p* < 0.05, CRUSH+(4.5 mg/kg)FB vs. SHAM and CRUSH; * indicates *p* < 0.01, SHAM vs. CRUSH and CRUSH+(1.5 mg/kg)FB; Ѱ indicates *p* < 0.05 CRUSH+(3 mg/kg)FB and CRUSH+(4.5 mg/kg)FB vs. SHAM; ^α^ *p* < 0.05, CRUSH+(3 mg/kg)FB vs. CRUSH and CRUSH+(1.5 mg/kg)FB.

Immediately in week 1the SHAM animals scored 2 out of 3 in the foot position test but restored the complete foot position score within week 2 (Figure 1A). Sciatic nerve crush injury showed a significant (*p* < 0.001) foot position deficit in the CRUSH group at week 1, week 2, week 3, and week 4 post-surgery compared to the SHAM group. All FB treatment doses displayed good recovery in the foot positioning test within week four compared to the CRUSH group. However, animals treated with 3 mg/kg and 4.5 mg/kg FB showed a significant (*p* < 0.01) increase in foot positioning scores compared to the 1.5 mg/kg FB treated group at week 4. There was no significant difference between the 3 mg/kg and 4.5 mg/kg FB treated groups compared to the SHAM group at week 4.

The SHAM animals showed minimal displacement in the toe spread test at week 1 (Figure 1B). However, the toe spread function was fully recovered within week 2. FB-treated groups showed faster recovery in the toe spread test compared to the CRUSH group. However, this improvement was only evident and significant in week 4. There was a significant improvement in the toe spread in CRUSH+(3 mg/kg) FB treated group (*p* < 0.01) and CRUSH+(4.5 mg/kg) FB-treated group (*p* < 0.05) compared to the CRUSH group within week 4.

The EPT test results showed immediate and significant (*p* < 0.001) recovery in the SHAM group compared to the CRUSH, CRUSH+(1.5 mg/kg) FB-treated, CRUSH+(3 mg/kg) FB-treated, and CRUSH+(4.5 mg/kg) FB-treated groups by week 1 till the day of sacrifice (Figure 1C). However, SHAM animals did not recover completely, indicating remaining minimal muscle injury influencing EPT outcome. Conversely, significant (*p* < 0.01) functional motor deficit was shown in the CRUSH group in week 2, 3, and 4 compared to CRUSH+(3 mg/kg) and CRUSH+(4.5 mg/kg) FB-treated groups. The (4.5 mg/kg) FB-treated group displayed faster recovery than the other FB-treated groups. In addition, it demonstrated a significant (*p* < 0.001) decrease in the functional motor deficit by week 3 and 4 compared to CRUSH animals. However, all FB-treated animals showed improvement in the EPT test at week 4 compared to the CRUSH animals.

The hopping test which examines several integrated sensory and motor functions demonstrated significant (*p* < 0.001) recovery in the SHAM animals compared to other groups at weeks 1 and 2 (Figure 1D). All FB-treated groups showed no hopping reflex within weeks 1 and 2. At week 3 post-injury, CRUSH and CRUSH + (1.5 mg/kg) FB treated group demonstrated a hopping response. However, a significant (*p* < 0.05) increase in the hopping reflex of the CRUSH+(3 mg/kg) FB and CRUSH+(4.5 mg/kg) FB-treated groups was noted in week 4 compared to the CRUSH group.

The rotarod test which evaluates the motor coordination of the rat displayed a significant (*p* < 0.001) decrease in the motor coordination of the CRUSH group and FB-treated groups compared to the SHAM group in week 1 post-surgery (Figure 1E). In week 2 post sciatic nerve crush injury, (3 mg/kg) and (4.5 mg/kg) FB-treated groups demonstrated significant (*p* < 0.05) improvement in motor coordination compared to the SHAM group. Meanwhile, the CRUSH group did not show any significant improvement in the speed of the rotarod test as compared to their performance on week 1 post-surgery. Within week 4, there was no significant difference between the rotarod speeds of (4.5 mg/kg) FB-treated animals and SHAM animals.

**FIGURE 2:**
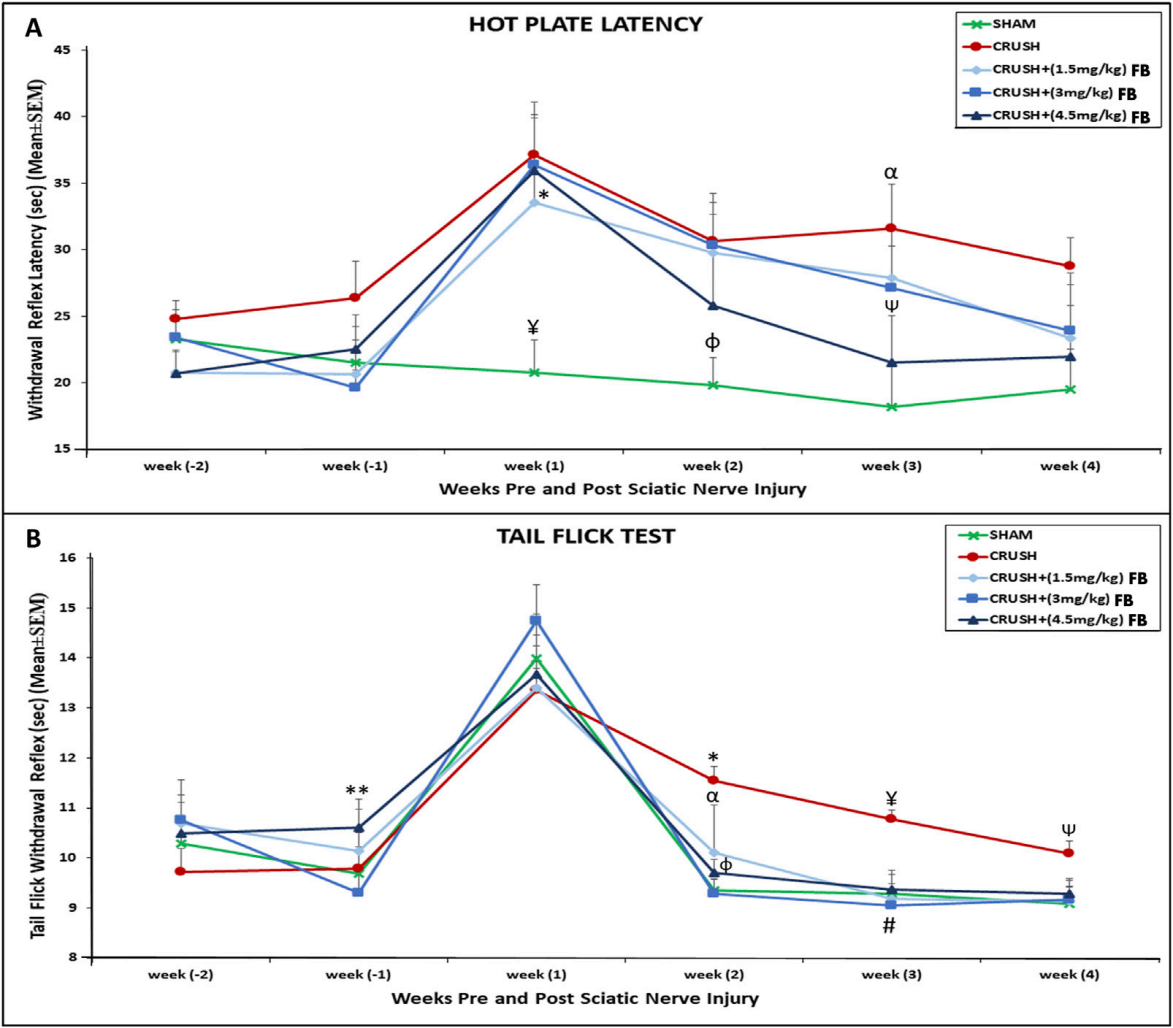
**(A)** Time course of hot plate latency. Nerve crush injury produced a severe nociception deficit in the Crush group. ¥ indicates *p* < 0.01, SHAM vs. CRUSH, CRUSH+(3 mg/kg) FB and CRUSH+(4.5 mg/kg) FB; * indicates *p* < 0.05, CRUSH+(1.5 mg/kg) FB vs. SHAM; φ indicates *p* < 0.05, SHAM vs. CRUSH and CRUSH+(3 mg/kg) FB; αindicates *p* < 0.01, CRUSH vs. SHAM; Ѱ indicates *p* < 0.05, CRUSH+(4.5 mg/kg) FB vs. CRUSH. **(B)** Time course of tail flick withdrawal latency. The treated group showed significant nociceptive recovery compared with the CRUSH group.*indicates *p* < 0.01, CRUSH vs. SHAM and CRUSH+(3 mg/kg) FB; α indicates *p* < 0.05, CRUSH+(1.5 mg/kg) FB vs. CRUSH; φ indicates *p* < 0.05, CRUSH+(4.5 mg/kg) FB vs. CRUSH; ¥ indicates *p* < 0.01, CRUSH vs. SHAM, CRUSH+(1.5 mg/kg) FB and CRUSH+(4.5 mg/kg) FB; # indicates *p* < 0.001, CRUSH+(3 mg/kg) FB vs. CRUSH; Ѱ indicates *p* < 0.05, CRUSH vs. SHAM and CRUSH+(1.5 mg/kg) FB; ** indicates *p* < 0.05, CRUSH+(4.5 mg/kg) FB vs. CRUSH+(1.5 mg/kg) FB.

The thermal hyperalgesia withdrawal reflex latency of CRUSH and FB-treated animals was significantly (*p* < 0.01) higher compared to SHAM animals at week 1 post-injury (Figure 2A). CRUSH rats did not show significant improvement in nociception withdrawal latency after sciatic nerve crush injury. The CRUSH group demonstrated significant (*p* < 0.01) higher nociception latency compared to the SHAM group at week 3. However, treatment with all FB doses prevented thermal hyperalgesia. There was no significant difference between all FB treatment doses and they were comparable to SHAM animals at week 4.

The tail-flick withdrawal reflex latencies showed no significant difference in all groups on week 1 post-surgery (Figure 2B). However, SHAM and CRUSH+(3 mg/kg) FB-treated groups displayed significant (*p* < 0.01) recovery in tail-flick latency. In comparison, CRUSH+(1.5 mg/kg) and CRUSH+(4.5 mg/kg) FB-treated groups demonstrated significant (*p* < 0.05) recovery in tail-flick latency compared to the CRUSH group at week 2. By week 4, all FB-treated and the SHAM groups completed full tail-flick withdrawal reflex recovery. In contrast, CRUSH animals did not achieve normal tail-flick withdrawal reflex compared to their performance on week 1 post-surgery.

### FB improves axonal regeneration recovery after sciatic nerve crush injury

#### Visualization of axonal regeneration using whole-mount staining of the crushed sciatic nerve

**FIGURE 3:**
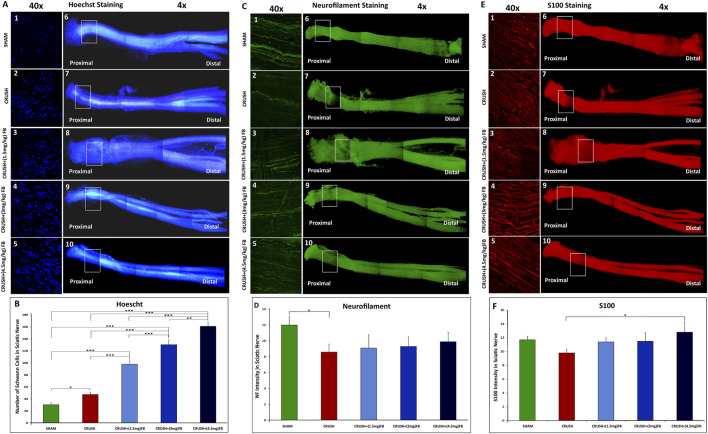
**(A–F)**: Visualization of axonal regeneration using whole-mount staining of the crushed sciatic nerve. **(A)** Comparison of the number of individual SC nuclei among experimental groups**. (A1-5)** representative images of Hoechst stained nuclei located at the crush injury site magnified from **(A6-10)** representative images. **(B)** The graph shows the quantification of Hoechst-stained nuclei. The number of nuclei in the field was analyzed from three or four sections, and average data from five independent experimental groups were compared. Data are expressed as mean ± SEM (n = 8). *p < 0.05, **p < 0.01, ***p < 0.001. **(C)** Re-growing axons of the sciatic nerve. The sciatic nerve is stained with neurofilament antibody 4 weeks after nerve crush injury **(C6-10).** At this point, more regenerating axons have grown within the FB-treated groups **(C3-5)** compared to the crush group **(C2). (D)** Neurofilament intensity was quantified in the graph using 4 different sections of the crush injury site. Data are expressed as mean ± SEM (n = 8). *p < 0.05. **(E)** Expression of S100 in SCs after 4 weeks from crush sciatic nerve injury. Images **(E 6-10)** represent whole-mount sciatic nerve preparations at the crush injury site. Higher magnification of crush site in **(E 1-5)** shows S100 positive SCs. **(F)** S100 intensity was quantified in the graph using 4 different sections of the crush injury site. Data are expressed as mean ± SEM (n = 8). *p < 0.05.

Whole-mount staining protocol was used to assess the extent of axonal regrowth and functional recovery following crush injury using neurofilament heavy chain antibody (NF), S100β antibody (S100), and counterstained with Hoechst dye. Figure 3A 1-10, 3C 1-10 and 3E 1-10) show an example of a preparation stained at week 4 following sciatic nerve crush injury. The site of the nerve crush injury can be easily distinguished by fluorescent microscopy using a ×4 magnifying objective (crush point marked by the square). The Hoechst stain may also identify the crush site, where the proliferation of SCs can be seen at the crush point (Dun and Parkinson, 2015). Neurofilament was used to stain the regenerating axons through the sciatic nerve, while S100β was used to stain the cytoplasm of SCs.

SCs are actively involved in regeneration during the process of peripheral axonal regeneration. SCs dedifferentiate, proliferate, and migrate toward the tips of regenerating axons, promoting axonal regeneration (Namgung, 2014; Kristján et al., 2015). Visualization of SCs nuclei in the crush area was done to compare the involvement of SCs among different experimental groups (Figure 3B). The number of nuclei was largely increased (*p* < 0.000) in CRUSH+(4.5 mg/kg) FB treatment as compared to SHAM, CRUSH, and CRUSH + (1.5 mg/kg) groups. CRUSH+(3 mg/kg) and CRUSH+(1.5 mg/kg) FB-treated animals also showed a significant increase (*p* < 0.000) in SCs proliferation as compared to SHAM and CRUSH animals. Schwann cells proliferation in the CRUSH group was high (*p* < 0.05) and incomparable to the SHAM group; however, the proliferation was low compared to FB-treated groups.

Regeneration patterns of sciatic nerve axons after the injury was investigated, and the labeling intensity of axons around the injury area was measured in all experimental groups (Figure 3C). A comparison of the intensity of NF in the crush injury site showed that axonal regeneration compared to the CRUSH group was gradually increased by FB treatment doses (1.5 mg/kg, 3 mg/kg, and 4.5 mg/kg) (Figure 3D). Injection of FB was effective in inducing axonal regrowth. However, the extent of axonal regrowth improvement was not significant (*p* > 0.05) at week 4. The intensity of labeled axons was markedly increased with CRUSH+(4.5 mg/kg) FB treatment.

To further investigate SCs function in axonal regeneration, we measured S100 staining intensity in the injured sciatic nerves (Figure 3E). At week 4 the labeling intensity of visualized SCs expressing S100 in the CRUSH+(4.5 mg/kg) FB treatment group was significantly higher (*p* < 0.05) as compared to the CRUSH group. In comparison, there was no significant difference between 1.5 mg/kg and 3 mg/kg FB-treated groups and the CRUSH group (Figure 3F). In addition, there was no significant difference in intensity levels between SHAM animals and CRUSH+(4.5 mg/kg) FB-treated animals at week 4.

**FIGURE 4:**
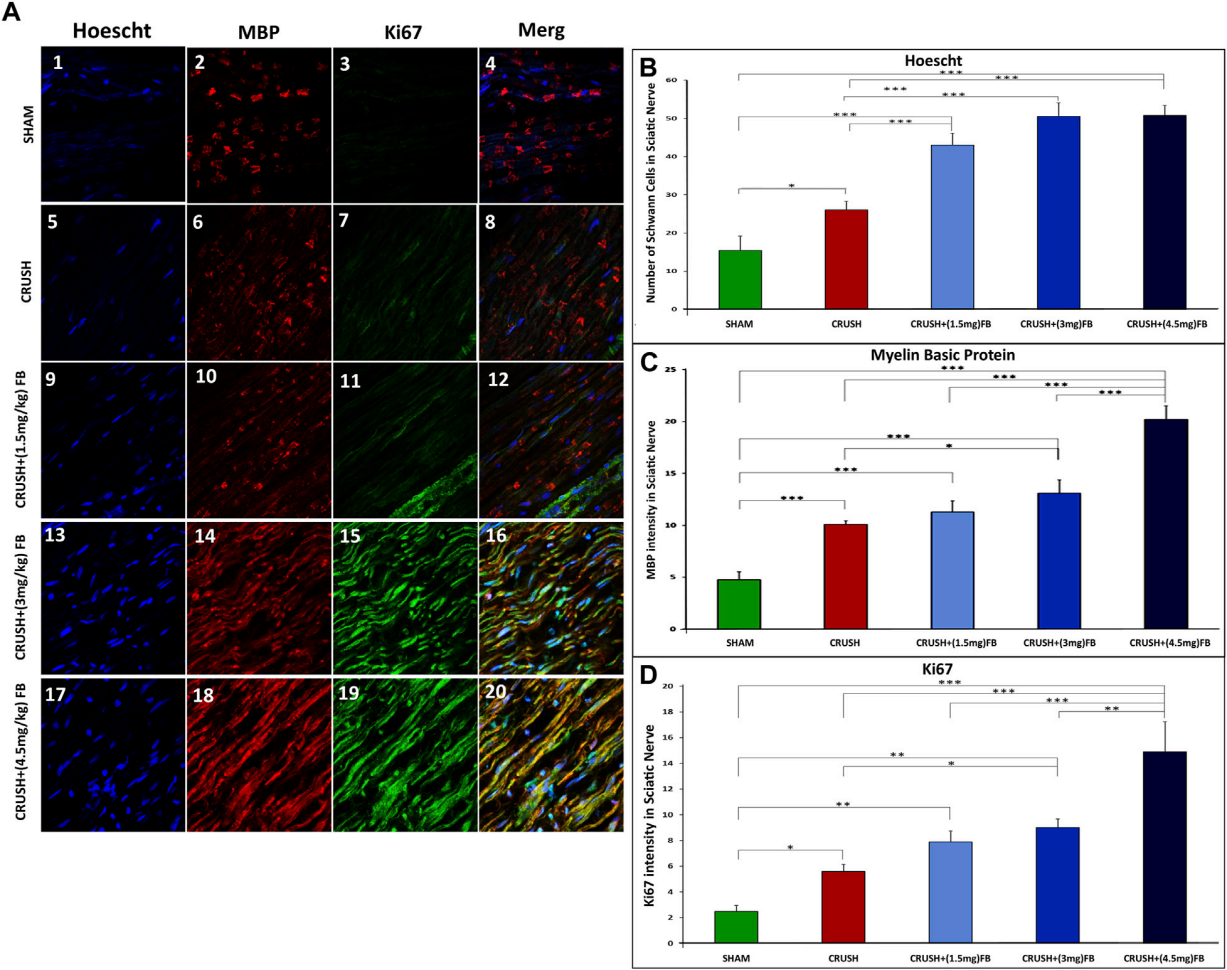
Whole-mount staining showing cell proliferation following sciatic nerve crush injury. Sciatic nerve was stained with myelin basic protein (MBP) in **(A 2, 6, 10, 14, and 18)** and Ki67 antibodies in **(A 3, 7, 11, 15 and, 19)** to show the presence of myelinated SCs and cell proliferation (Ki67) at 4 weeks after nerve crush injury. Nuclei are counterstained with Hoechst dye (Ho) in **(A 1, 5, 9, 13 and 17)**. **(B)** Quantification of the number of individual SC nuclei among different experimental groups. The number of nuclei from 4 different sections was analyzed, and average data from five independent experimental groups were compared. Data are expressed as mean ± SEM (n = 8). *p < 0.05, **p < 0.01, ***p < 0.001. **(C)** Quantification of MBP fluorescence intensity in different groups. MBP intensity was analyzed from 4 different sections, and average data from five independent experimental groups were compared. Data are expressed as mean ± SEM (n = 8). *p < 0.05, **p < 0.01, ***p < 0.001. **(D)** Quantification of Ki67 fluorescence intensity in different groups. The intensity of Ki67-expressed cells was analyzed from 4 different sections, and average data from five independent experimental groups were compared. Data are expressed as mean ± SEM (n = 8). *p < 0.05, **p < 0.01, ***p < 0.001.

To further demonstrate the whole-mount staining protocol following nerve crush injury, we also stained nerve preparations with myelin basic protein (MBP) and Ki67 to assess cell proliferation and myelinated SCs within the regenerating nerve at week 4 after crush injury. Figure 4A shows the proliferating Ki67-positive SCs within the nerve and MBP-positive myelin Sheaths. The count of SCs nuclei shown in Figure 4B was nearly the same in all FB-treated groups. However, these groups displayed a significant increase (*p* < 0.000) in SCs counts compared to the CRUSH and SHAM groups. Similarly, the data showed a highly significant increase (*p* < 0.000) of MBP intensity in the CRUSH+(4.5 mg/kg) FB treatment group as compared to the other groups (Figure 4C). CRUSH, CRUSH + (1.5 mg/kg), and CRUSH+(3 mg/kg) FB treatment groups demonstrated significant (*p* < 0.000) levels of MBP as compared to SHAM group. In addition, the CRUSH group showed elevated (*p* < 0.05) SCs counts compared to the SHAM group.

Ki67 was weakly detected in the intact sciatic nerve of the SHAM group (Figure 4D), but was strongly induced after injury. Ki67 intensity levels were significantly elevated (*p* < 0.000) in CRUSH+(4.5 mg/kg) FB treatment group in comparison to other groups. The intensity of Ki67 in CRUSH+(3 mg/kg) FB-treated animals showed a significant increase as compared to CRUSH animals (*p* < 0.05) and SHAM animals (*p* < 0.01). CRUSH and CRUSH+(1.5 mg/kg) FB-treated group also displayed a significant increase (*p* < 0.05, *p* < 0.01, respectively) in Ki67 intensity as compared to the SHAM group.

#### FB has neuroprotective effects on spinal cord neurons after sciatic nerve crush injury

**FIGURE 5:**
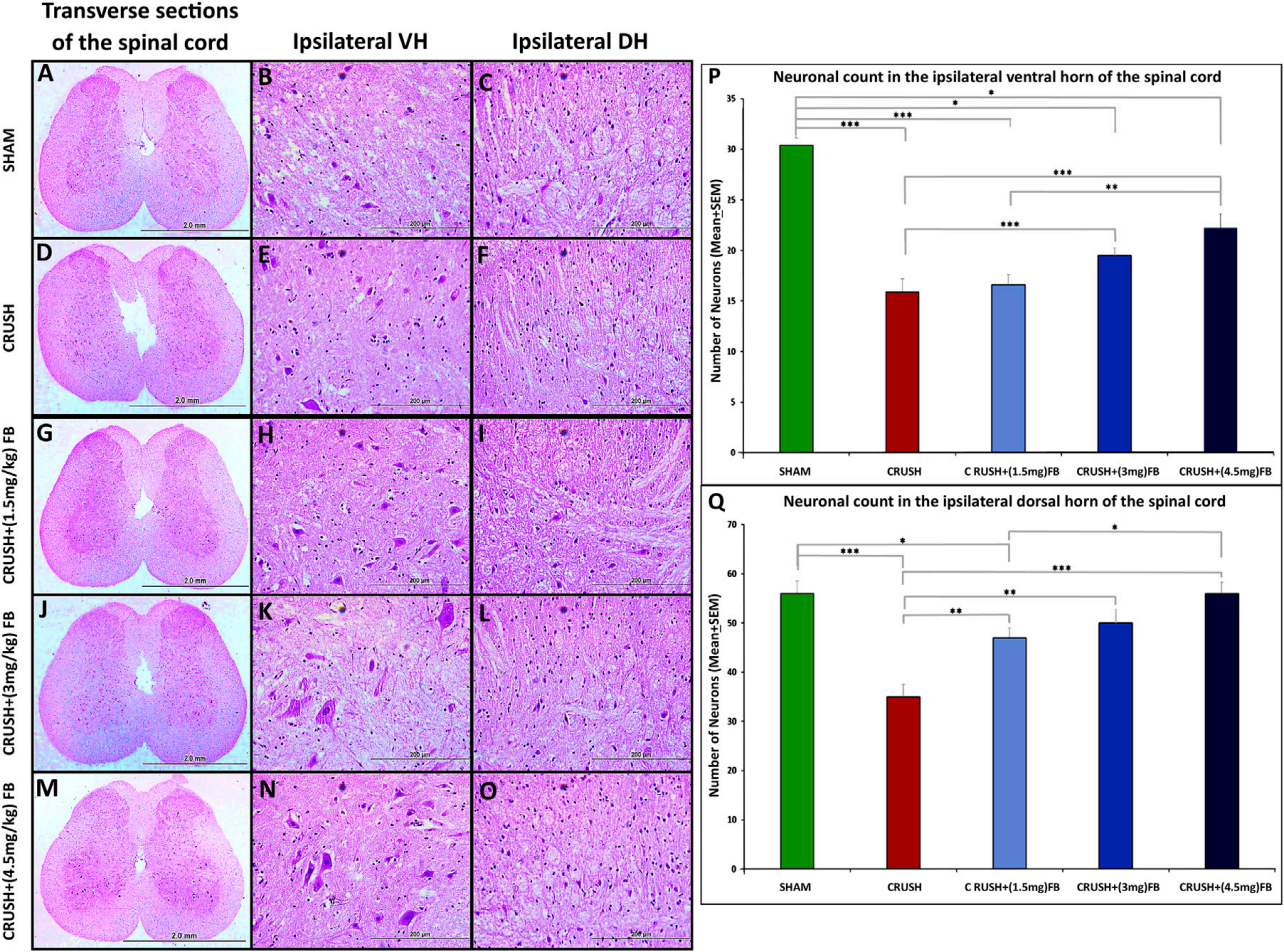
**(A–O)**: Representative photomicrographs (×4 and ×40 objectives) of Nissl staining of the ipsilateral ventral (VH) and dorsal horns (DH) of the lumbar spinal cord transverse section. SHAM group shows normal neurons in the ipsilateral ventral and dorsal horns **(B, C)**. Compared to the SHAM group, the CRUSH group displays irregular neurons with eccentric nuclei **(E, F)**. FB-treated groups **(G–O)** show protected neurons compared to the CRUSH group **(D–F)**. **(P)** Statistical analysis of the mean number of ventral horn motor neurons among the five experimental groups. CRUSH animals showed a reduced number of neurons after sciatic nerve crush injury. FB-treated groups displayed a significant increase in ventral horn neurons compared to the CRUSH group. Data are expressed as mean ± SEM. *p < 0.05, **p < 0.01, and ***p < 0.001. **(Q)** Statistical analysis of the mean number of dorsal horn sensory neurons among the five experimental groups. CRUSH animals showed a reduced number of neurons after sciatic nerve crush injury. FB-treated groups significantly increased dorsal horn sensory neurons compared to the CRUSH group. Data are expressed as mean ± SEM. *p < 0.05, **p < 0.01, and ***p < 0.001.

Histological analysis of Cresyl Violet stained sections of lumbar spinal cord ventral and dorsal grey horns at week 4 after sciatic nerve injury are shown in Figures 5A–O. The CRUSH animals revealed a remarkable reduction of observed healthy ipsilateral ventral and dorsal horn neurons as compared to the SHAM group at week 4 In addition, CRUSH animals’ spinal cord sections showed the absence of Nissl substance within the ipsilateral neurons of ventral and dorsal horns and the eccentrically displaced nucleus of ventral horn neurons. Morphometric analysis of the CRUSH group showed a significantly (*p* < 0.001) lower number of neurons in the ventral horn when compared to the SHAM, CRUSH+(3 mg/kg), and CRUSH+(4.5 mg/kg) FB-treated groups (Figure 5P). In addition, CRUSH+(4.5 mg/kg) FB-treated group demonstrated a significantly (*p* < 0.01) higher number of neurons when compared to CRUSH+(1.5 mg/kg) FB group. However, there was no significant difference in the neuronal count between CRUSH+(4.5 mg/kg) and CRUSH+(3 mg/kg) FB-treated groups. Correspondingly, the number of dorsal horn neurons in the FB-treated groups is more than in the CRUSH group at week 4 (Figure 5Q). In the dorsal horn, the CRUSH group displayed a significantly (*p* < 0.01 and *p* < 0.001) lower neuronal count when compared to CRUSH+(1.5 mg/kg), CRUSH+(3 mg/kg), and CRUSH + (4.5 mg/kg) FB-treated animals. Furthermore, CRUSH+(4.5 mg/kg) FB-treated group showed a significantly (*p* < 0.05) higher number of neurons when compared to the CRUSH+(1.5 mg/kg FB-treated group. In contrast, was no significant difference in dorsal horn neuronal count between CRUSH + (4.5 mg/kg) and CRUSH+(3 mg/kg) FB-treated groups.

**FIGURE 6:**
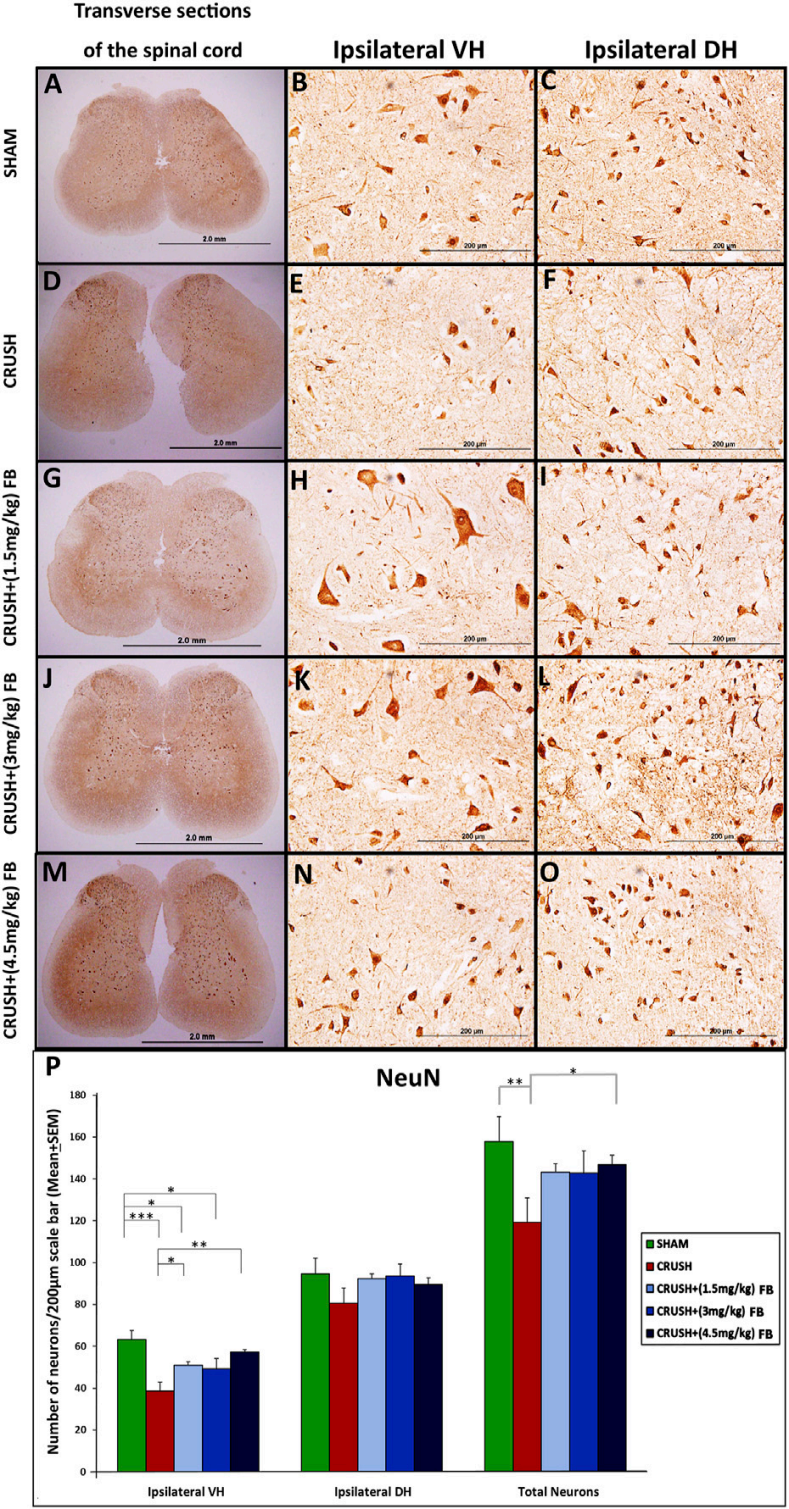
**(A–O)**: NeuN immunoreactivity staining in the ipsilateral lumbar spinal cord regions after sciatic nerve crush injury in all experimental groups **(A–O)**. NeuN-positive neurons are identified as brown cells (Magnification ×4 and 40x). **(P)** Means of labeled NeuN immunoreactive neurons/field in the spinal cord sections of rats subjected to sciatic nerve crush injury. NeuN neuronal count was less in the CRUSH group after sciatic nerve crush injury. There were no significant differences in neuronal loss in the FB-treated rats. Data are expressed as mean ± SEM. *p < 0.05, **p < 0.01, and ***p < 0.001.

Qualitative analysis of the NeuN immunostaining was performed on spinal cord sections obtained from all experimental groups (Figures 6A–O). NeuN-immunostained neurons were shown to be distributed in the grey matter of the lumbar spinal cord regions. Data revealed a noticeable difference between the CRUSH group and other experimental groups at week 4 post-injury. Further, quantitative analysis (Figure 6P) of NeuN-immunostained neurons showed a significant (*p* < 0.001) decrease in the number of the NeuN immunostained neurons in the CRUSH group as compared to SHAM at week 4 post-injury. (Further, the treated groups with (1.5 mg/kg) FB and (4.5 mg/kg) FB showed a significant (*p* < 0.05 and *p* < 0.01, respectively) increase in the number of NeuN positive neurons compared to the CRUSH group at week 4. However, there was no significant (*p* > 0.05) difference in the number of NeuN immunostained neurons between all FB-treated groups. Likewise, the quantitative analysis ofNeuN-immunostained neurons in the ipsilateral dorsal horn of the spinal cord revealed a decrease in the number of NeuN immunostained neurons in the CRUSH group (Figure 6P). Still, this decrease was not significant (*p* > 0.05) between all the experimental groups. Despite that, FB-treated groups displayed a higher number of NeuN immunostained neurons when compared with the CRUSH group. The total number of NeuN immunostained neurons was significantly (*p* < 0.05) higher in CRUSH + (4.5 mg/kg) FB-treated animals as compared to CRUSH animals. On the other hand, CRUSH animals demonstrated a significantly (*p* < 0.01) reduced number of the total ipsilateral NeuN immunostained neurons compared to the SHAM group.

### FB inhibits the crush sciatic nerve injury-induced neuronal and axonal apoptosis

#### Expression of c-Jun in ventral and dorsal horn neurons of the spinal cord after sciatic nerve crush injury

**FIGURE 7:**
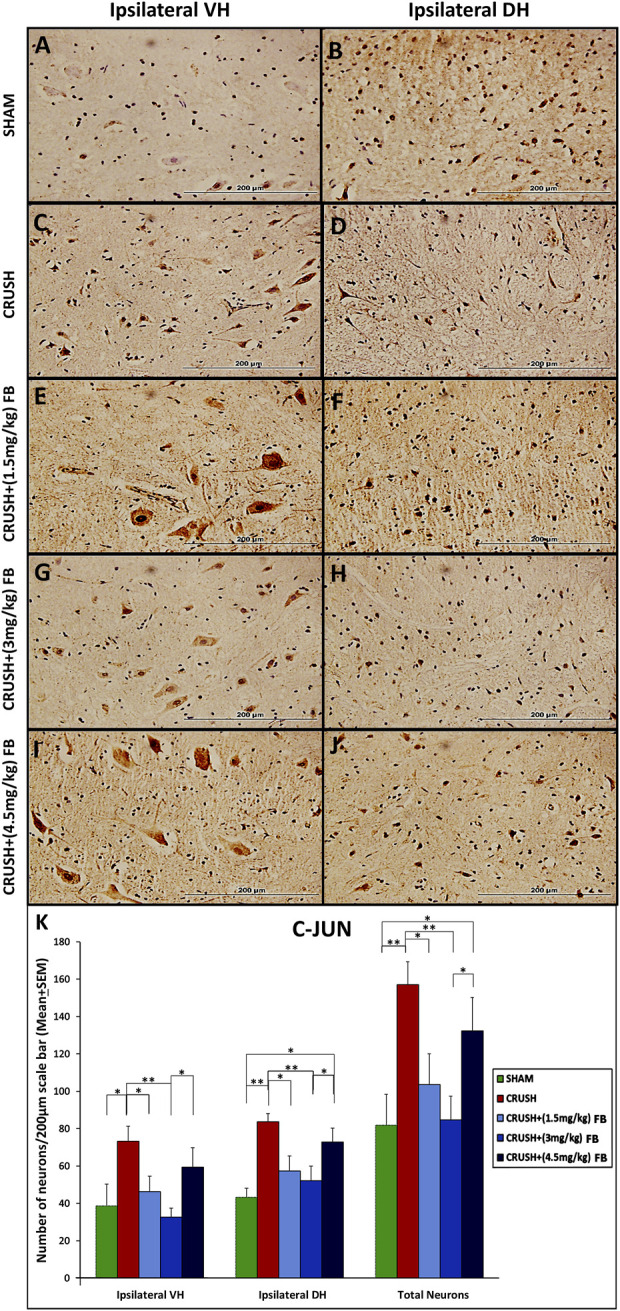
**(A–J)**: c-Jun immunoreactive neurons in ipsilateral lumbar spinal cord segments following sciatic nerve crush injury. C-Jun-positive neurons were observed with a brown-stained nucleus in the ipsilateral ventral and dorsal grey horns (magnification ×40). **(K)** Number of c-Jun-positive neurons at week 4 after sciatic nerve crush surgery. The numbers of c-Jun-positive neurons increased significantly in the spinal cord’s ventral and dorsal horns in all the experimental groups following sciatic nerve crush injury compared with those in the sham-operated group. Data are expressed as mean ± SEM. *p < 0.05, **p < 0.01, and ***p < 0.001.

c-Jun protein was expressed in ventral and dorsal horn neurons after sciatic nerve crush injury (Figure 7). The staining of c-Jun was exclusively neuronal (Figures 7A–J).and predominantly nuclear at the cellular level; however, faint staining was observed in the cytoplasm of the cell body and neuronal processes. At week 4, the c-Jun immunoreactivity in ipsilateral ventral horn neurons of the spinal cord was significantly (*p* < 0.05) higher in the CRUSH group as compared to the SHAM group (Figure 7K). Analysis of c-Jun immunoreactive neurons in both ventral and dorsal horns revealed that c-Jun in treated animals with (1.5 mg/kg) FB and (3 mg/kg) FB were significantly lower (*p* < 0.05 and *p* < 0.01, respectively) than the CRUSH group. CRUSH + (4.5 mg/kg) FB-treated animals demonstrated significantly higher c-Jun immunoreactive neurons in ventral and dorsal horns than (3 mg/kg) in FB-treated animals. Also, CRUSH+(4.5 mg/kg) FB-treated group showed significantly (*p* < 0.05) higher expression of the c-Jun protein in dorsal horn neurons as compared to the SHAM group.

#### Expression of c-Fos in ventral and dorsal horn neurons of the spinal cord after sciatic nerve crush injury

**FIGURE 8:**
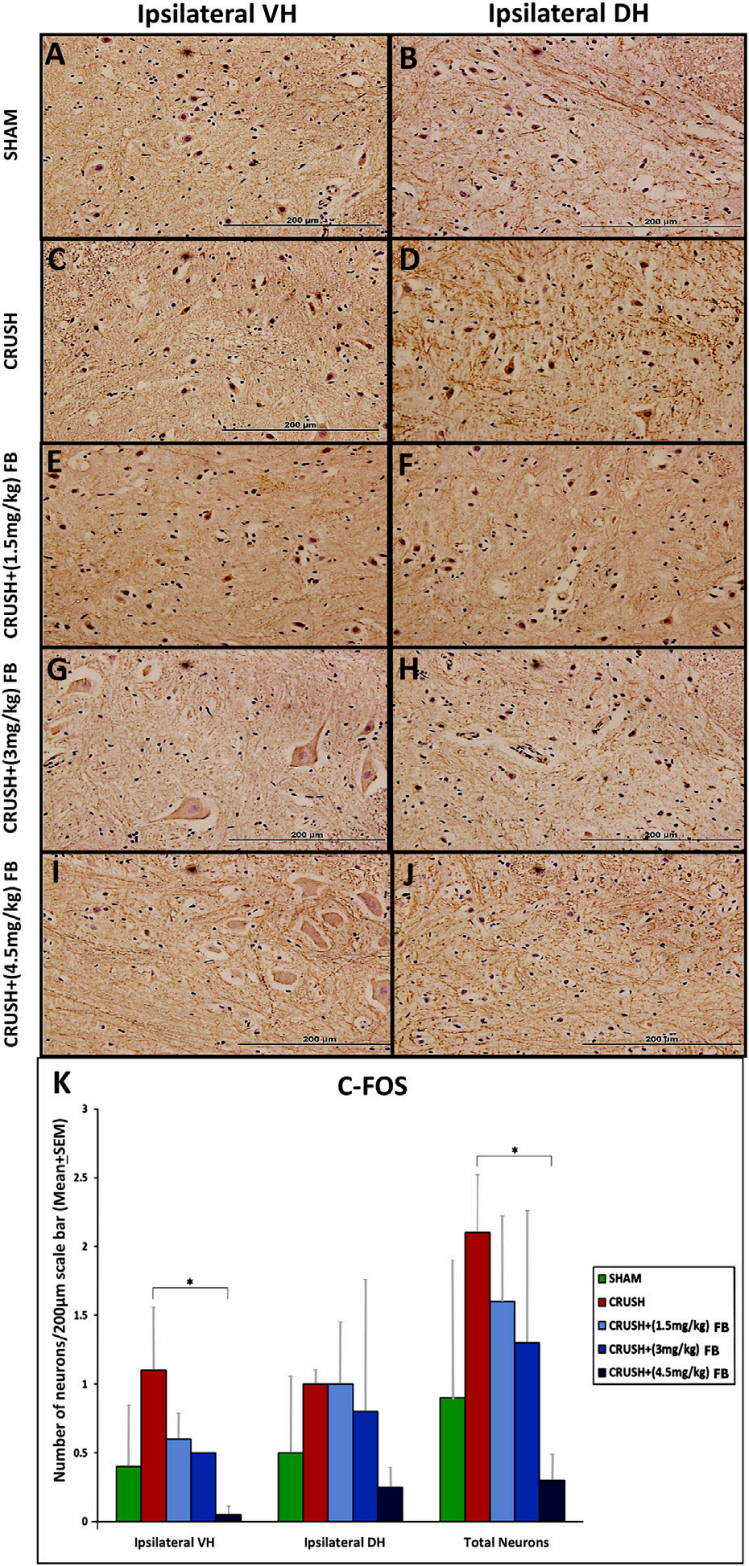
**(A–J)**: c-Fos immunohistochemistry in the ipsilateral lumbar spinal cord regions after sciatic nerve crush injury. Weak immunoreactivity was observed in the nuclei of the spinal neurons of the CRUSH group (Magnification ×40). **(K)** After sciatic nerve crush injury, the number of c-Fos positive neurons in the spinal cord’s ipsilateral ventral and dorsal horns. The number of c-Fos positive neurons markedly decreased with FB treatment in the spinal cord’s anterior and dorsal horns. Data are expressed as mean ± SEM. *p < 0.05, **p < 0.01, and ***p < 0.001.

Expression of c-Fos was induced in spinal cord neurons after sciatic nerve crush injury (Figure 8). Immunostaining was restricted to the nucleus within the neuron as in c-Jun immunostaining (Figures 8A–J). However, the expression of c-Fos was feeble at week 4 due to the downregulation of the protein. Few c-Fos expressed neurons were scattered in the grey horn at week 4 post-injury. Analysis of c-Fos immunoreactive neurons revealed significantly (*p* < 0.05) higher expression of the protein in the ventral horn of crush group as compared to the CRUSH + (4.5 mg/kg) FB-treated group (Figure 8K). There was no significant difference between the other experimental groups as compared to each other.

#### Expression of 8-OHdG (oxidative DNA damage marker) in spinal cord neurons after sciatic nerve crush injury

**FIGURE 9:**
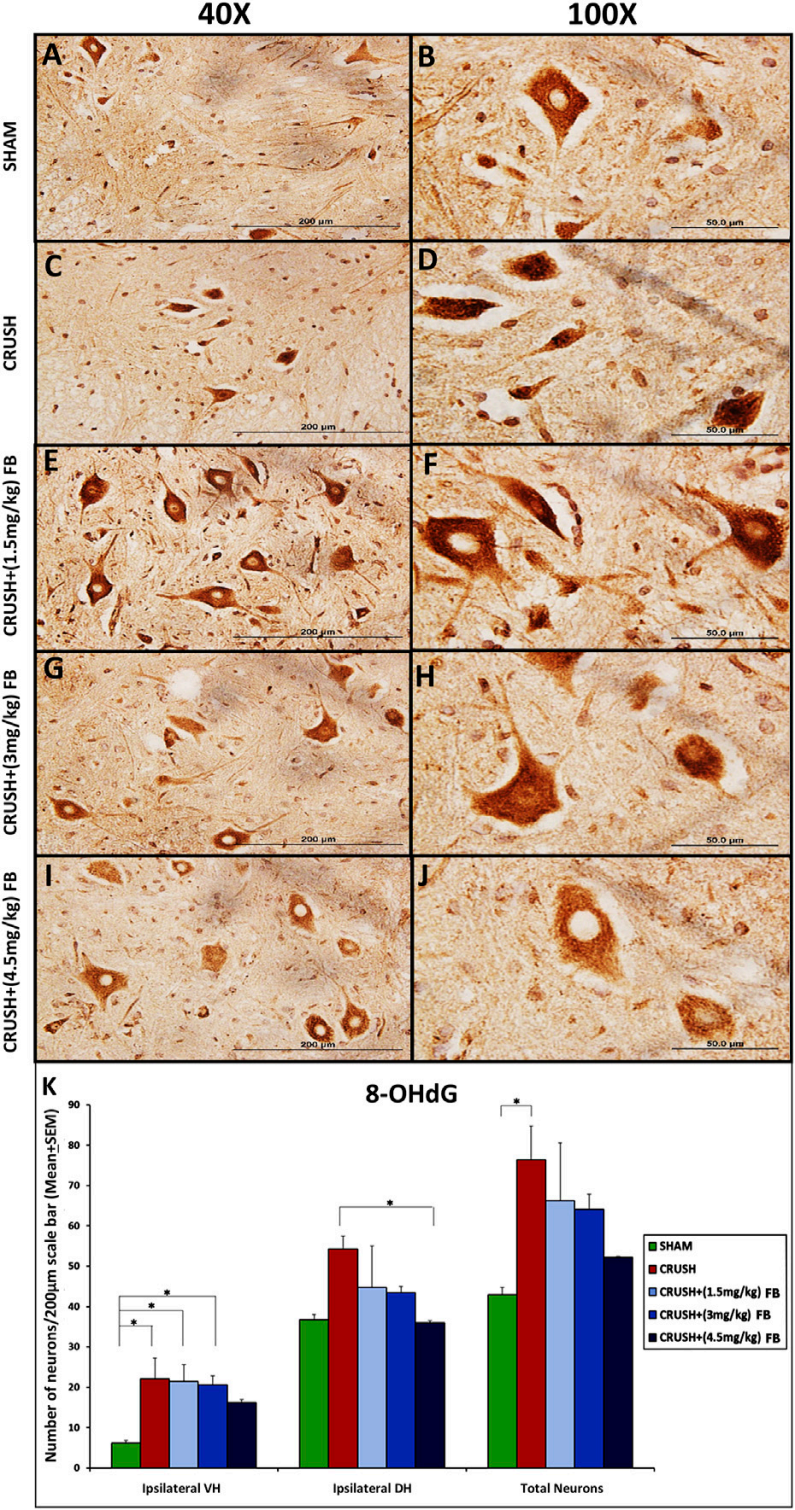
**(A–J)**: Representative immunohistochemical staining of 8-OHdG in the ventral horn segment of the spinal cord after sciatic nerve crush injury. 8-OHdG positive neurons were identified with brown nuclei among all experimental groups (Magnification ×40 and 100x). **(K)** After sciatic nerve crush injury, the number of positive neurons for 8-OHdG in the spinal cord’s ipsilateral ventral and dorsal horns. 8-OHdG positive neurons were highly identified in the ventral horn of the spinal cord in the CRUSH group after injury. FB-treated groups showed lower 8-OHdG expression in the spinal cord when compared to the CRUSH group. Data are expressed as mean ± SEM. *p < 0.05, **p < 0.01, and ***p < 0.001.

Elevated levels of neurons expressing 8-OHdG were observed in the CRUSH group’s ventral horn of the spinal cord (Figures 9A–J). Ipsilateral ventral horn neurons of the spinal cord demonstrated significantly (*p* < 0.05) higher levels of 8-OHdG in the CRUSH group as compared to the SHAM group (Figure 9K). CRUSH + (1.5 mg/kg) and CRUSH+(3 mg/kg) FB-treated groups revealed significantly (*p* < 0.05) elevated levels of the 8-OHdG marker in ventral horn neurons as compared to the SHAM group. CRUSH + (4.5 mg/kg) FB-treated animals showed no significant (*p* > 0.05) difference in 8-OHdG protein levels as compared to the SHAM group. In ipsilateral dorsal horn neurons, CRUSH animals showed significantly (*p* < 0.05) higher levels of 8-OHdG protein as compared to the CRUSH + (4.5 mg/kg) FB-treated group. The total number of 8-OHdG expressing neurons was significantly (*p* < 0.05) higher in CRUSH animals as compared to SHAM animals (Figure 9K).

#### Neuronal cell death in spinal cord neurons after sciatic nerve crush injury

**FIGURE 10:**
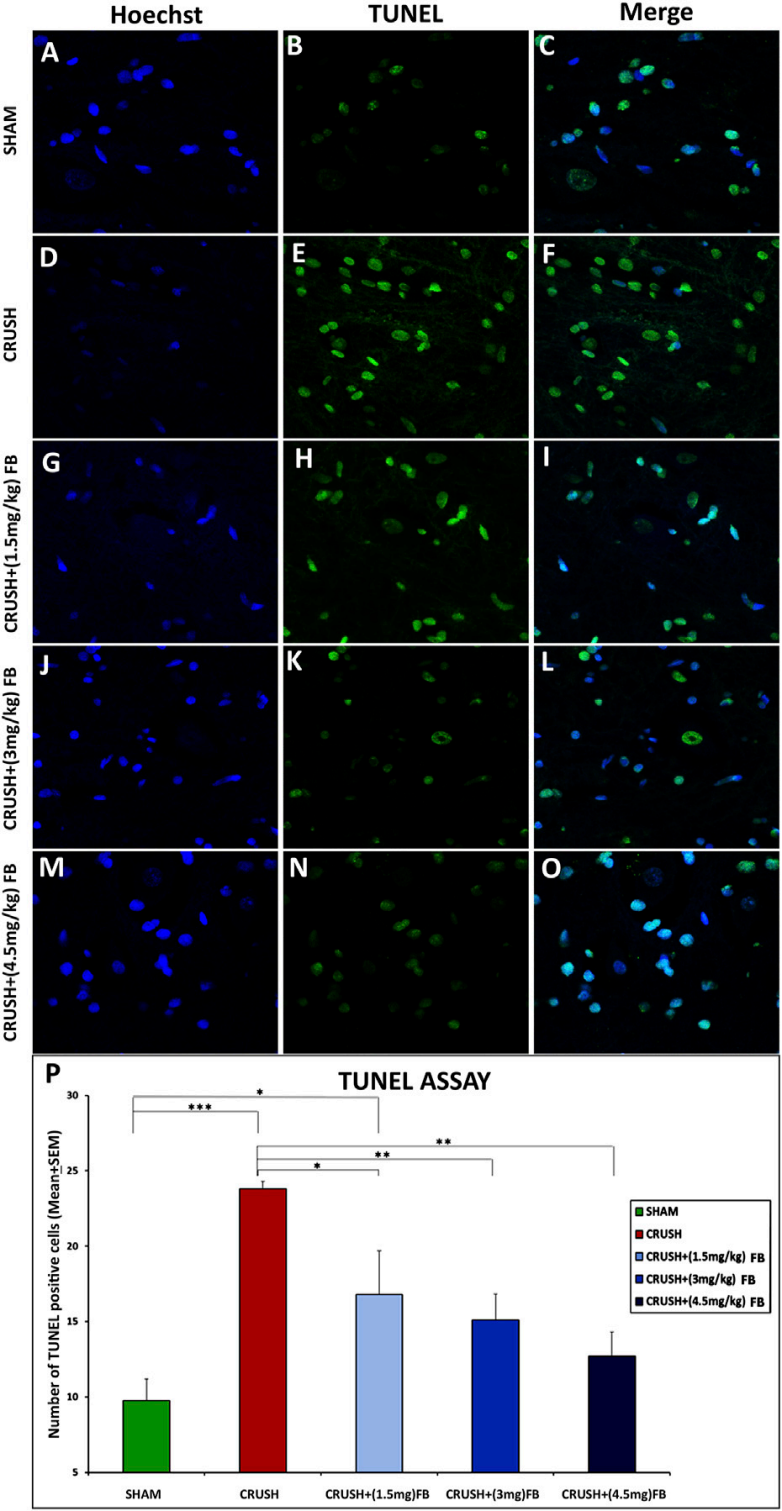
FB reduced apoptosis after sciatic nerve crush injury. The apoptotic cells of the spinal cord were identified through a TUNEL assay. **(B, E, H, K, and N)** Cells with a TUNEL signal are presented in (green) and counterstained with Hoechst in **(A, D, G, J and M)** (blue). **(C,F,I,L,O)** photos representing merged Hoechst and TUNEL immunopfluorescence staining Magnification ×40. **(P)** The number of TUNEL-positive cells within the spinal cord. CRUSH animals demonstrated a higher number of TUNEL-positive cells when compared to other experimental groups. FB treatment significantly decreased the number of apoptotic cells after sciatic nerve crush injury. Data are expressed as mean ± SEM. *p < 0.05, **p < 0.01, and ***p < 0.001.

TUNEL-positive cells were rarely detected in the SHAM group, while sciatic nerve crush injury caused extensive cell death of spinal cord neurons (Figure 10A–O). There were significantly (*p* < 0.001) more TUNEL-positive cells in the CRUSH group than in the SHAM group (Figure 10P). All FB-treated groups (1.5 mg/kg, 3 mg/kg, and 4.5 mg/kg) demonstrated significantly (*p* < 0.05, *p* < 0.01, and *p* < 0.01, respectively) fewer TUNEL positive cells as compared to the CRUSH group at week 4 post-injury. CRUSH + (1.5 mg/kg) FB-treated animals showed significantly (*p* < 0.05) higher TUNEL-positive cells than SHAM animals. On the other hand, there was no difference between the numbers of TUNEL-positive cells between FB-treated group doses (Figure 10P).

#### FB inhibits cytochrome c release and caspase-3 activation after sciatic nerve crush injury

**FIGURE 11:**
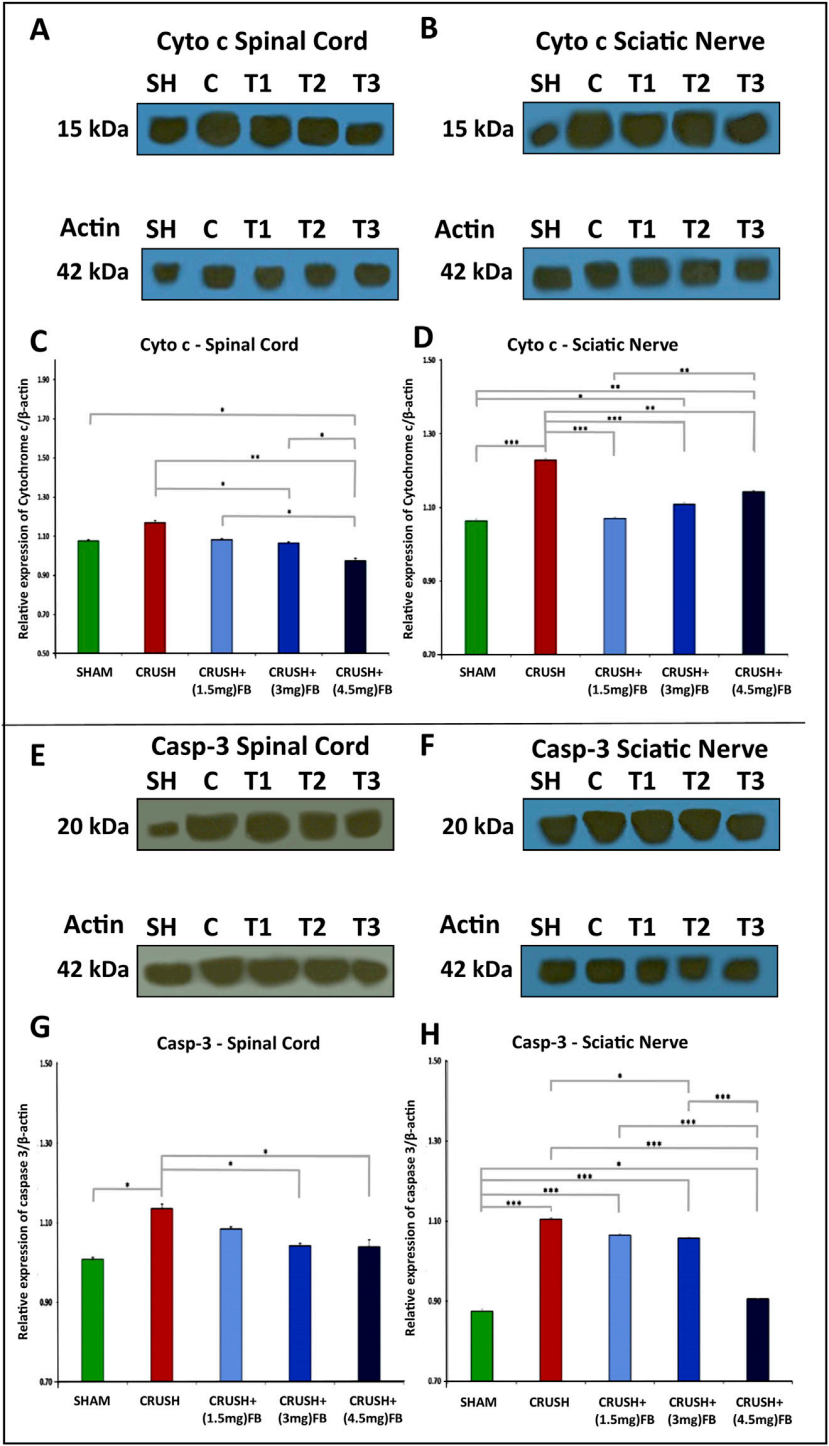
**(A–D):** FB inhibits cytochrome c release after sciatic nerve crush injury. After the injury, spinal cord samples at week 4 after injury were processed for Western blot analysis. Western blot analysis of cytochrome c with cytoplasmic fractions in the spinal cord **(A and C)** and sciatic nerve **(B and D)** samples after injury. **(C, D)** Quantitative Western blotting analysis shows that FB treatment significantly decreased cytochrome c release into cytoplasm compared with the CRUSH group after injury. Data are expressed as mean ± SEM. *p < 0.05, **p < 0.01, and ***p < 0.001.**(E–H):** FB inhibits caspase-3 activation after sciatic nerve crush injury. Western blot analysis of activated caspase-3 in the spinal cord **(E and G)** and sciatic nerve **(F and H)** samples after injury. **(C, D)** Quantitative Western blotting analysis **(C, D)** shows that the active caspase-3 was increased in the CRUSH group after sciatic nerve crush injury. FB treatment significantly decreased the level of activated caspase-3 compared with the CRUSH group after injury. Data are expressed as mean ± SEM. *p < 0.05, **p < 0.01, and ***p < 0.001.

The release of mitochondrial cytochrome c and the activation of caspase-3 after sciatic nerve crush injury occur at an early stage of apoptotic cell death (Figure 11). Western blot analysis revealed that cytochrome c release into the cytoplasm increased in the spinal cord and sciatic nerve samples after week 4 in crush groups (Figures 11A–D). The expression of released cytochrome c was significantly (*p* < 0.001) lower in CRUSH + (1.5 mg/kg) and CRUSH+(3 mg/kg) FB-treated groups compared to the CRUSH group in spinal cord samples (Figures 11A,C). However, CRUSH + (4.5 mg/kg) FB-treated group demonstrated significantly (*p* < 0.001) lower expression of cytochrome c protein than the CRUSH group and SHAM group (*p* < 0.05) in spinal cord samples. Also, CRUSH + (4.5 mg/kg) FB-treated animals showed a significant (*p* < 0.001) decrease in the level of cytochrome c as compared to other FB-treated animals in spinal cord samples. In contrast, post-treatment with FB doses (1.5 mg/kg, 3 mg/kg, and 4.5 mg/kg) significantly (*p* < 0.01-*p*<0.001) decreased the mitochondrial release of cytochrome c compared to the CRUSH group in sciatic nerve samples (Figures 11B,D). In sciatic nerve tissue, CRUSH+(4.5 mg/kg) FB-treated group displayed significantly (*p* < 0.01) increased levels of cytochrome c compared to the CRUSH+(1.5 mg/kg) FB-treated group. In comparison, there was no significant difference in cytochrome c protein levels between CRUSH + (4.5 mg/kg) and CRUSH + (3 mg/kg) FB-treated groups.

Likewise, the activated forms of caspase-3 increased in spinal cord and sciatic nerve after crush injury compared to the SHAM group (Figures 11E–H). Furthermore, CRUSH+(3 mg/kg) and CRUSH+(4.5 mg/kg) FB treatments significantly (*p* < 0.05) decreased the level of caspase-3 activation when compared with the CRUSH animals in spinal cord samples (Figures 11E,G). There was no significant difference between the CRUSH+(1.5 mg/kg) FB treatment group and the CRUSH group in the level of activated caspase-3 protein in sciatic nerve samples (Figures 11F,H). On the other hand, CRUSH+(3 mg/kg) FB-treated animals demonstrated significantly (*p* < 0.05) lower levels of caspase-3 as compared to CRUSH animals in sciatic nerve samples. CRUSH+(4.5 mg/kg) FB-treated group showed significantly (*p* < 0.001) decreased levels of activated caspase-3 as compared to CRUSH, CRUSH+(1.5 mg/kg), and CRUSH + (3 mg/kg) FB-treated groups in sciatic nerve samples (Figure 11H). These results indicated that FB treatment inhibits cytochrome c release and caspase-3 activation in the spinal cord and sciatic nerve after sciatic nerve crush injury.

#### FB treatment reduces the pro-apoptotic protein bax and increases the anti-apoptotic protein Bcl-2

**FIGURE 12:**
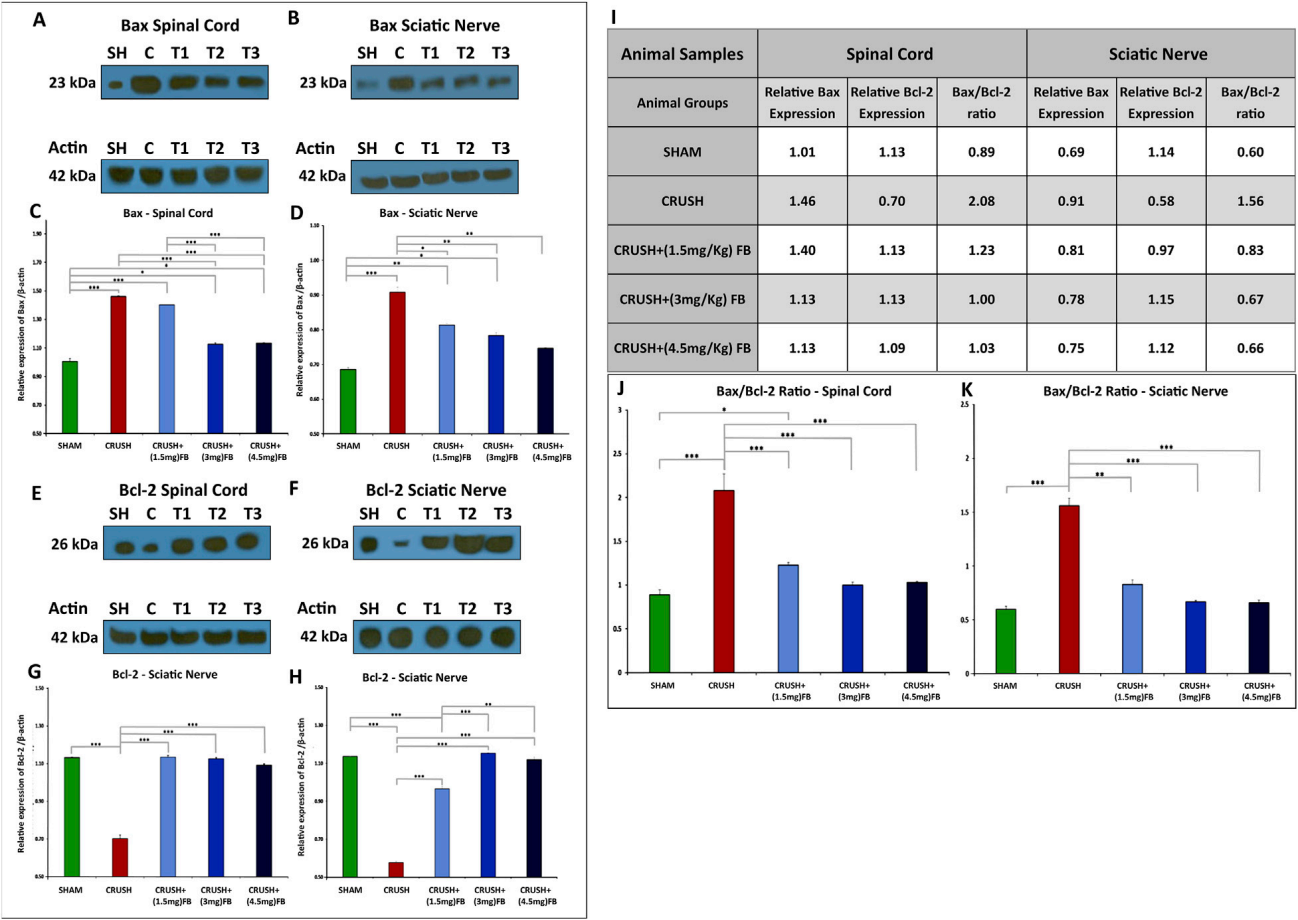
**(A–D):** FB inhibits neuronal apoptosis after sciatic nerve crush injury. The expression levels of Bax in the spinal cord neurons **(A and C)** and sciatic nerve **(B and D)** were detected using Western blot analysis at week 4 after sciatic nerve crush injury. **(C, D)** Quantitative analysis of Western blotting shows increased Bax expression levels in the CRUSH group after injury. However, Bax expression levels were significantly decreased in FB treatment groups compared to the CRUSH group. Data are expressed as mean ± SEM. *p < 0.05, **p < 0.01, and ***p < 0.001.**(E–H):** FB inhibits neuronal apoptosis after sciatic nerve crush injury. The expression levels of Bcl-2 in the spinal cord neurons **(E and G)** and sciatic nerve **(F and H)** were detected using Western blot analysis at week 4 after sciatic nerve crush injury. **(G, H)** Quantitative analysis of Western blotting shows decreased Bcl-2 expression levels in the CRUSH group after injury. However, Bcl-2 expression levels were significantly increased in FB treatment groups compared to the CRUSH group. Data are expressed as mean ± SEM. *p < 0.05, **p < 0.01, and ***p < 0.001. **(I–K):** The Bax/Bcl-2 ratio in the spinal cord neurons and sciatic nerve was detected using Western blot analysis at week 4 after sciatic nerve crush injury. **(I)** Comparison of Bax/Bcl-2 ratio on spinal cord and sciatic nerve crush injury after FB treatment. **(J, K)** Quantitative analysis shows decreased Bax/Bcl-2 ratio in spinal cord and sciatic nerve in all FB-treated groups compared to the CRUSH group after injury. Data are expressed as mean ± SEM. *p < 0.05, **p < 0.01, and ***p < 0.001.

Western blot analysis revealed that Bax protein (Figures 12A–D) is markedly upregulated and Bcl-2 protein (Figures 12E–H) is down-regulated in the spinal cord and sciatic nerve samples after sciatic nerve crush injury. However, after sciatic nerve crush injury, FB administration significantly reduced the expression levels of Bax and upregulated Bcl-2 expression. CRUSH+(3 mg/kg) and CRUSH+(4.5 mg/kg) FB-treated groups demonstrated significantly (*p* < 0.001) decreased Bax expression levels compared to CRUSH group and CRUSH+(1.5 mg/kg) FB-treated group in spinal cord samples (Figures 12A,C). There were no significant differences in Bax levels between CRUSH compared to CRUSH+(1.5 mg/kg) FB-treated group and CRUSH+(3 mg/kg) FB-treated group compared to CRUSH+(4.5 mg/kg) FB-treated group in the spinal cord samples (Figures 12A,C). Likewise, in sciatic nerve tissue, the expression of Bax protein in the CRUSH+(1.5 mg/kg) FB-treated group was significantly (*p* < 0.05) lower when compared to the CRUSH group (Figures 12B,D). Also, CRUSH+(3 mg/kg) and CRUSH+(4.5 mg/kg) FB-treated groups demonstrated significantly (*p* < 0.01) reduced levels of Bax protein compared to the CRUSH group. However, there was no significant difference between all FB treatment doses in Bax protein expression. Despite that CRUSH+(4.5 mg/kg) FB-treated group showed a lower expression level of Bax protein (Figures 12B,D). Similarly, the expression of the anti-apoptotic protein Bcl-2 was significantly (*p* < 0.001) decreased in the CRUSH group as compared to all experimental groups in both spinal cord and sciatic nerve samples (Figures 12E–H). CRUSH+(1.5 mg/kg) FB-treated group showed significantly reduced Bcl-2 expression levels as compared to CRUSH+(3 mg/kg) (*p* < 0.001) and CRUSH+(4.5 mg/kg) (*p* < 0.01) FB-treated groups in sciatic nerve samples (Figure 12H). There was no significant difference between CRUSH+(3 mg/kg) and CRUSH+(4.5 mg/kg) FB-treated groups in the expression of Bax protein levels in sciatic nerve samples (Figure 12H). The calculated Bax/Bcl-2 ratios in the spinal cord and sciatic nerve crush injury after FB treatment are displayed in Figure 12I. Quantitative analysis (Figures 12J,K) shows decreased Bax/Bcl-2 ratio in spinal cord and sciatic nerve in all FB-treated groups compared to the CRUSH group.

## Discussion

### Sciatic nerve crush injury model, FB administration time, and dose selection for FB treatment

The present study investigated several functional and biochemical parameters to address the effect of FB derived from CSP on peripheral nerve regeneration, neural protection, and functional recovery after sciatic nerve crush injury. Sciatic nerve crush injury is the most widely used model for peripheral nerve injury (Kirsch et al., 2009; Ngeow et al., 2011; Noorafshan et al., 2011). This animal model is well established to study the efficacy of new therapies for accelerating and improving axon growth and regeneration after injury. Easy access to the sciatic nerve is one advantage of making it a method of choice as a model compared to other peripheral nerve injuries. Due to sciatic nerve crush injury, a sequence of degenerative processes occur distal to the injury site combined with specific histological changes at the injury site or proximal to it (Mietto et al., 2011).

The first 2 weeks after sciatic nerve crush injury are the most crucial period of the neurodegeneration process. After that, the inflammatory response, degradation of myelin, removal of myelin debris, and SCs activity occur. Therefore, the time frame selected in this study for FB treatment duration was appropriate to affect these processes and protect the neurons of the spinal cord and SCs in the sciatic nerve from the apoptotic process (Griffin et al., 2013; Wood and Mackinnon, 2015; Li et al., 2020; Jiang et al., 2021). This present study and our previous studies have started FB treatment immediately following sciatic nerve crush injury and lasted for 2 weeks to minimize the degeneration process and enhance the process of regeneration, which begins at the end of the second week after the crush injury. Recently, we have shown that sciatic nerve crush injured animals treated with a dose of 3 mg/kg FB (IP) showed significant neurobehavioral and histological improvements (Renno et al., 2021). In this study, FB was administered (IP) after sciatic nerve crush injury at a dose level of (1.5 mg/kg), (3 mg/kg), and (4.5 mg/kg). Animals did not show any significant changes in body weight among the experimental groups during the study (data not shown). Neither side effects nor mortality was observed with treatment with these doses of FB.

### Neurotherapeutics and regenerative effects of FB treatment on sciatic nerve crush injury model

In the present study, IP treatment with FB improved the performance of different sensory and motor neurobehavioral functions in rats after sciatic nerve crush injury. Loss of motor and sensory functions in the CRUSH group was an indication of the success of producing the sciatic nerve injury model as well as consistency with the previous work done in our laboratory Renno et al., 2013; Renno et al., 2017; Renno et al., 2021). Treatment with FB showed enhancement in sensory function compared to the CRUSH group. This included improving the tail-flick and hot plate tests after FB treatment. Likewise, FB-treated rats improved motor recovery as evaluated by hopping, rotarod, and EPT tests. Also, the toe spread and foot positioning tests were improved with FB treatment compared to the CRUSH group. The neurobehavioral data were supported by nerve visualization using axonal markers through whole-mount staining to study the axonal regeneration, SCs proliferation, and SC-axon interaction in the peripheral nerve following injury (Namgung, 2014; Dun and Parkinson, 2015). Neurofilament (NF) is a component of the neuronal cytoskeleton, playing a role in maintaining neuronal stability. Loss of this stability may lead to neurodegenerative diseases (Wang et al., 2012). FB treatment caused sciatic nerve axonal regeneration to some extent, but was not significant compared with the CRUSH group in neurofilament labeling. Since the animals were sacrificed on the 28th day post sciatic nerve crush injury, the nerves did not have sufficient time to show significant changes in the NF staining after the injury.

The SCs are actively involved in the peripheral axonal regeneration process, where they dedifferentiate, proliferate, and migrate to the tip of re-growing axons, promoting axonal regeneration (Jessen et al., 2015). FB treatment enhanced SCs proliferation, resulting in SCs responses as evidenced by Hoechst, S100, and Ki67 labeling. Also, labeling MBP in the sciatic nerve indicated a significant increase in its expression in FB-treated animals, consistent with our previous biochemical work (Renno et al., 2021). Taken together, FB appears to be involved in enhancing axonal recovery by acting on SCs in the injured axons. FB is a mixture of biochemically and pharmacologically active components that effectively treat diabetic neuropathy and chronic back and joint pain (Al-Hassan, 1990). However, the pathophysiologic principle of how FB works on the nervous system is still unknown. Further, our present study indicates that SCs in regenerating nerves could be another target of FB Fraction B treatment, upregulated the expression of Ki67 in myelinated SCs. Ki67 is a protein associated with cell proliferation, and it is detected within the nucleus during the interphase, whereas it is relocated at the surface of the chromosomes during mitosis (Scholzen and Gerdes, 2000). Expression levels of Ki67 in SCs were moderate with (1.5 mg/kg) and (3 mg/kg) FB treatments when compared to that in the SHAM group but strongly upregulated with (4.5 mg/kg) FB treatment. Collectively, these results suggest that FB treatment may induce Ki67 expression in SCs that controls growth cone guidance at the tip of re-growing axons.

We further enhanced the neurobehavioral recovery and nerve visualization data with histological and immunohistological analysis of spinal cord neurons. It is well known that significant neuronal cell death and axon degeneration occur after peripheral axonal injury, resulting in functional deficits (Watkinsa et al., 2013; Renno et al., 2015; Renno et al., 2017; Renno et al., 2021). Consistent with our previous studies, CRUSH animals demonstrated a significant reduction in the number of spinal cord ipsilateral ventral and dorsal horn neurons compared to SHAM and FB-treated groups (Renno et al., 2015; Renno et al., 2017; Renno et al., 2021). In this study, histological examination of Cresyl violet stained sections of the ventral and dorsal horns of the lumbar spinal cord showed that CRUSH animals revealed less number of healthy ventral and dorsal horn neurons. On the other hand, FB-treated animals demonstrated higher numbers of neurons in the ventral and dorsal horns of the spinal cord. Also, the number of NeuN-immunoreactive neurons in the ventral and dorsal horn of the spinal cord was significantly more in FB-treated groups compared to the CRUSH group at week 4 post-injury. These findings revealed that FB treatments remarkably alleviated the neurodegenerative modifications seen in Cresyl violet-stained neurons of the ventral and dorsal horn of the lumbar spinal cord. Furthermore, as stated in our previous studies, FB treatment strongly prevented the decrease in the number of NeuN immunostained spinal cord neurons in the crush sciatic nerve animal model.

Recently, several growth factors were discovered in the protein fraction of FB, including nerve growth factors and human and rat stem cell factors (Jassim M. Al-Hassan, unpublished data). In light of these findings, the nerve growth factor and stem cell factors may play a major role in the neuronal and glial neuroregeneration reported in this study and previously (Renno et al., 2021). The lipid fraction contains furan fatty acids (F-Acids), among which the F6 was the most studied F-acid. Recently, our collaborators have shown that this F-Acid acts as an anti-cancer, anti-inflammatory, and antioxidant, causes NETosis and is involved in wound healing. Another active lipid is cholesta-3,5-diene (S5), which is anti-inflammatory and promotes wound healing (Al-Hassan et al., 2019; Al-Hassan et al., 2020). Cholesta-3,5,6- triol was also found in the lipid fraction of FB (Al-Hassan et al., 2022). It acted as a potent anti-cancer compound. The role of the growth factors, especially the stem cell and nerve growth factors and the active lipids found in FB in the case of nerve regeneration has not been established. This will be the subject of future studies.

### Anti-apoptotic effects of FB treatment on sciatic nerve crush injury model

This study demonstrates that c-Jun expression at the protein level increases in the neurons of the ventral and dorsal horns of the spinal cord in the CRUSH and CRUSH + FB treated groups. Expression of c-Jun was presented in apoptotic cells, giving evidence to its association with cell death. Furthermore, this expression was associated with apoptotic morphology, where the neurons of the CRUSH group were presented with shrinkage and a peripherally located nucleus. The expression of c-Jun was not exclusively for the nucleus. It was also observed in the cytoplasm, suggesting a role of c-Jun in apoptotic cell death, as stated previously (Ferrer et al., 1996). However, the association of c-Jun and apoptotic morphology was occasional with FB treatment. This difference indicates that c-Jun expression is not restricted to cell death. Most likely that c-Jun expression in CRUSH + FB treated animals is related to another cellular response. It is tempting to suppose that the increased expression of c-Jun with FB treatment in this study is highly associated with neuronal survival and regeneration since there were no apparent signs of degeneration. Previous studies demonstrated that axonal sprouting response and the ability of the neuron to survive are associated with increased c-Jun levels, as c-Jun has a role in the regeneration response (Haas et al., 1996; Herdegen et al., 1997). Therefore, the increased c-Jun expression in FB-treated groups may be implicated in the regenerative responses to injury. In this regard, further investigations are needed to support this postulation.

In contrast to c-Jun, the c-Fos expression was exclusively in the nucleus of the spinal cord neurons, indicating that induced apoptotic cell death occurs. However, c-Fos expression was feeble at week 4 post sciatic nerve crush injury. Also, we did not observe any co-localization of c-Fos staining and apoptotic morphology in FB-treated groups. One reason is that c-Fos is an early gene highly expressed immediately after noxious stimuli (Bullitt et al., 1992). Still, by the time we sacrificed the animals at week 4, c-Fos was downregulated or no longer expressed. Virtually, there was no induction of c-Fos immunoreactivity at a cellular level in FB-treated groups. Therefore, we favor the interpretation that c-Fos expression does not play a role in the nerve regeneration process at week 4 following crush injury. Its role remains to be further investigated in the early phase post-nerve injury.

Oxidative damage to DNA causes various kinds of damage in the cell (Mecocci et al., 1998). The biomarker 8-OHdG has been used to measure DNA damage due to the hydroxyl radical attack at the C8 position of the nucleobase guanine or its nucleoside guanosine (Valavanidis et al., 2009). This study has shown that neurons of the ventral and dorsal horns of the spinal cord show significant 8-OHdG immunoreactivity following sciatic nerve crush injury, suggesting that neurons are subjected to oxidative DNA damage. After the injury, this damage may result from excitotoxic mechanisms leading to cellular DNA, mitochondrial instability, and modification errors. The level of the 8-OHdG biomarker was highly expressed in the CRUSH group that showed apoptotic changes, while it was significantly reduced with FB treatment. The increase of 8-OHdG immunoreactivity after sciatic nerve crush injury indicates its oxidative DNA damaging effects because of the high amounts of free radical formation and antioxidant inhibition. Therefore, our results suggest that oxidative DNA damage in the spinal cord neurons was ameliorated by FB treatment after sciatic nerve crush injury Fraction B may neutralize reactive oxygen species produced after injury, suggesting that the DNA damage repair mechanism needs to be supported by further studies.

Apoptosis is an active process that occurs after inducing external or internal stimuli to different cells. During this process, shrinkage occurs in cells forming apoptotic cell bodies that enclose the cleaved DNA that can be utilized by TUNEL assay to confirm the presence of apoptosis after injury. Further, apoptosis includes different morphological and biochemical changes. The most constant change during apoptosis is activating an endonuclease that results in DNA cleavage at the internucleosomal liner sites. This, in turn, allows the TUNEL assay TdT to attach to the free OH-DNA fragments in the apoptotic cell (Lou et al., 1998). This study’s visualization of TUNEL-stained apoptotic cells revealed significantly higher numbers of TUNEL-positive nuclei in the CRUSH group. On the other hand, the number of TUNEL-positive nuclei was significantly decreased with FB treatment. The knowledge that FB alleviates apoptotic cell death after sciatic nerve crush injury may give additional strategies for limiting neuronal cell death, thus improving neurological functions.

We have demonstrated for the first time that FB treatment of crushed-injured sciatic nerves improved functional recovery by alleviating apoptotic cell death of spinal cord neurons. We have also shown that the neuroregenerative effect of FB on the sciatic nerve might be mediated in part by increasing the proliferation rate of SCs after injury. Finally, we demonstrated that FB alleviated oxidative DNA damage after sciatic nerve crush injury. We further supported these data biochemically by using Western blotting techniques for apoptotic and anti-apoptotic proteins. The primary molecular proteins involved in the regulation of apoptosis are caspases, Bax, and Bcl-2. The suppression of neuronal cell apoptosis is required to achieve functional and histopathological recoveries following nerve injury (Tang et al., 2014; Zhang et al., 2015; Chen et al., 2016). Bax and Bcl-2 are the major members of Bcl-2 family that play a crucial role in promoting or inhibiting intrinsic apoptotic pathway triggered by mitochondrial dysfunction (Mohan et al., 2012). Bax promotes cell death through the permeabilization of mitochondrial outer membrane in response to different cellular stresses. In contrast, Bcl-2 prevents apoptosis by inhibiting the activity of Bax. After the neuronal injury, the pro-apoptotic proteins Bax, caspase-9, and caspase-3 are upregulated, while the anti-apoptotic protein Bcl-2 is downregulated (Yuan and Yankner, 2000). Our results indicated that the anti-apoptotic effect of FB could be mediated through the mitochondrial-dependent apoptotic pathway because treatment with FB inhibited the release of cytochrome c and the activation of caspase-3 in the spinal cord and sciatic nerve. Similarly, FB treatment significantly down-regulated Bax protein expression in the spinal cord and sciatic nerve but significantly upregulated the expression level of Bcl-2 in these tissues.

The equilibrium balance between pro- and anti-apoptotic components of Bcl-2 family can determine the cellular destiny (Yip and Reed, 2008). Further, down-regulation and up-regulation of Bax and Bcl2 expressions was shown to be associated with better survival in many diseases, respectively (Zeestraten et al., 2013). Increased Bax/Bcl-2 ratio up-regulates caspase-3 and increases apoptosis in many diseases such as in the thymus of patients with myasthenia Gravis (Salakou et al., 2007), human osteoarthritis (Georgios et al., 2015), bladder cancer (Matsumoto et al., 2004) and colorectal cancer (Khodapasand et al., 2015).

## Conclusion

The present study investigated the anti-apoptotic and regenerative effects of FB on the sciatic nerve crush injury model. Based on the results above and our other published work, we concluded that FB does not produce any side effects or reduction in the bodyweight of the rats. Further, FB improves the performance of rats on different sensory and motor neurobehavioral functional tests and induces nerve axonal regeneration and recovery by acting on SCs and injured axons following the sciatic nerve crush injury. Also, FB alleviates the neurodegenerative changes in neurons of the ventral and dorsal horn of the lumbar spinal cord by its anti-apoptotic property and recovers the decrease in the neuronal count of ventral and dorsal horns of the lumbar spinal cord following sciatic nerve crush injury. In addition, FB inhibits DNA damage in spinal cord neurons, reduces neuronal cell death resulting from sciatic nerve crush injury, and improves neurological functions. In conjunction with our recently published data (Renno et al., 2021), these results help explain the therapeutic trial successes using FB prepared from catfish epidermal secretions on patients with chronic severe back and joint pain. Our results makes FB a candidate for translational studies for application to treatment of nerve injuries and possibly chronic untreatable degenerative diseases such as Parkinson’s and Alzheimer’s.

## Data Availability

The raw data supporting the conclusion of this article will be made available by the authors, without undue reservation.
